# CalmBelt: Rapid SARS-CoV-2 Genome Characterization for Outbreak Tracking

**DOI:** 10.3389/fmed.2021.790662

**Published:** 2021-12-14

**Authors:** Hatairat Yingtaweesittikul, Karrie Ko, Nurdyana Abdul Rahman, Shireen Yan Ling Tan, Niranjan Nagarajan, Chayaporn Suphavilai

**Affiliations:** ^1^Advanced Research Center for Computational Simulation, Faculty of Science, Chiang Mai University, Chiang Mai, Thailand; ^2^Department of Mathematics, Faculty of Science, Chiang Mai University, Chiang Mai, Thailand; ^3^Genome Institute of Singapore, A*STAR, Singapore, Singapore; ^4^Department of Microbiology, Singapore General Hospital, Singapore, Singapore; ^5^Department of Molecular Pathology, Singapore General Hospital, Singapore, Singapore; ^6^Duke-NUS Medical School, Singapore, Singapore; ^7^Yong Loo Lin School of Medicine, National University of Singapore, Singapore, Singapore

**Keywords:** SARS-CoV-2, whole genome sequencing, outbreak tracking, nanopore sequencing, web application

## Abstract

**Background:** The ongoing COVID-19 pandemic is a global health crisis caused by the spread of SARS-CoV-2. Establishing links between known cases is crucial for the containment of COVID-19. In the healthcare setting, the ability to rapidly identify potential healthcare-associated COVID-19 clusters is critical for healthcare worker and patient safety. Increasing sequencing technology accessibility has allowed routine clinical diagnostic laboratories to sequence SARS-CoV-2 in clinical samples. However, these laboratories often lack specialized informatics skills required for sequence analysis. Therefore, an on-site, intuitive sequence analysis tool that enables clinical laboratory users to analyze multiple genomes and derive clinically relevant information within an actionable timeframe is needed.

**Results:** We propose CalmBelt, an integrated framework for on-site whole genome characterization and outbreak tracking. Nanopore sequencing technology enables on-site sequencing and construction of draft genomes for multiple SARS-CoV-2 samples within 12 h. CalmBelt's interactive interface allows users to analyse multiple SARS-CoV-2 genomes by utilizing whole genome information, collection date, and additional information such as predefined potential clusters from epidemiological investigations. CalmBelt also integrates established SARS-CoV-2 nomenclature assignments, GISAID clades and PANGO lineages, allowing users to visualize relatedness between samples together with the nomenclatures. We demonstrated multiple use cases including investigation of potential hospital transmission, mining transmission patterns in a large outbreak, and monitoring possible diagnostic-escape.

**Conclusions:** This paper presents an on-site rapid framework for SARS-CoV-2 whole genome characterization. CalmBelt interactive web application allows non-technical users, such as routine clinical laboratory users in hospitals to determine SARS-CoV-2 variants of concern, as well as investigate the presence of potential transmission clusters. The framework is designed to be compatible with routine usage in clinical laboratories as it only requires readily available sample data, and generates information that impacts immediate infection control mitigations.

## Introduction

The ongoing coronavirus disease (COVID-19) pandemic, caused by the betacoronavirus SARS-CoV-2, is a rapidly evolving global health crisis. As of 13th September 2021, more than 224 million cases of COVID-19 resulting in more than 4.6 million deaths have occurred worldwide (1, 2). In Singapore, 71,687 COVID-19 cases have been identified since the beginning of the pandemic (3). The aggressive contact tracing and quarantine efforts had helped to reduce the spread of SARS-CoV-2 in Singapore (4).

The sequencing of SARS-CoV-2 from clinical samples is crucial for the understanding and control of SARS-CoV-2. Genomic surveillance of SARS-CoV-2 enables the detection and close monitoring of emergent variants which may be associated with increased transmissibility or disease severity, or which may impair the effectiveness of current vaccines, diagnostic methods, therapeutics, and public health control strategies. Emerging SARS-CoV-2 variants are closely monitored by WHO (5) and various health authorities (6, 7), emphasizing the importance of genomic surveillance. Additionally, whole-genome comparative analysis of SARS-CoV-2 from clinical samples provides valuable transmission information to guide clustering of linked COVID-19 cases (8). This is particularly useful where epidemiological data alone is insufficient to link cases due to human and circumstantial factors. Clustering of linked cases facilitates the use of contact tracing and quarantine measures to limit the spread of SARS-CoV-2.

Since the first SARS-CoV-2 whole-genome sequences were made available on the sequence repository GISAID (9) in January 2020, over 3.4 million SARS-CoV-2 sequences have been shared from around the world. The availability of low cost handheld sequencers, such as Oxford Nanopore Technologies (ONT) (10), has significantly lowered entry and sequencing costs, allowing many laboratories to sequence SARS-CoV-2 from clinical samples. These laboratories can share and analyse the SARS-CoV-2 whole-genome sequences at the regional and population level via GISAID (9) and genomic analytic platforms such as Nextstrain (8), allowing large-scale pathogen evolution tracking.

However, barriers remain in place that limit the ability of these sentinel laboratories to obtain meaningful analysis of small sets of locally sequenced SARS-CoV-2 genomes for the purposes of local outbreak investigations. One key barrier is that diagnostic laboratories often lack specialized informatics skills for the analysis of whole-genome sequence data. This results in delays to analysis of the data thus obtained.

Another barrier in place is that the PANGO lineage nomenclature (11) currently used for the classification of SARS-CoV-2 outbreaks does not provide the resolution required for local outbreak investigations. The PANGO lineage assigned to a SARS-CoV-2 whole-genome sequence is based on a large phylogenetic tree of SARS-CoV-2 genomes. As it is not practical to rebuild the large phylogenetic tree whenever new genomes become available, the new genomes are classified into different subtrees (lineages) according to the prediction from a machine learning model, which has been regularly trained to predict lineage using whole genome information. However, the performance of the machine learning model (decision tree) is not perfect, with a macro-average precision of 91.66% and a recall of 91.51% across 778 pre-defined PANGO lineages (11, 12). Additionally, some pre-defined sets of mutations (decision tree rules) for classifying PANGO lineages are limited. For instance, only 79 genomic loci [with 12 mutations (13)] were chosen by the decision tree for classifying lineage B.1.617.2 (14).

To address these barriers, we propose CalmBelt, an integrated framework for on-site whole-genome characterization and outbreak tracking. Compared to existing analytic frameworks such as GISAID and Nextstrain (8, 9), CalmBelt addresses the issue of resolution required for local outbreak investigations by using data driven analysis based on whole-genome sequence and sample collection information as an alternative strategy to the pre-defined phylogenetic tree and machine learning model of the PANGO lineage. Furthermore, unlike available command-line bioinformatics tools (11, 15–17), CalmBelt provides an easy-to-use platform for sentinel clinical diagnostic laboratories to analyse whole-genome sequences on-site, as soon as they become available. The intuitive design of CalmBelt allows users to integrate sample collection date and pre-defined epidemiological data together with whole-genome sequence data. With just a few clicks, CalmBelt can provide a customized visualization of genome relatedness together with the established SARS-CoV-2 nomenclature of GISAID clades and PANGO lineages for each whole-genome sequence. Therefore, CalmBelt allows users to preliminarily and rapidly interrogate the locally generated set of whole-genome sequence data, negating the biohazard risks and delay associated with SARS-CoV-2 sample transfer, or delay associated with submission to a curated database such as GISAID (9).

We demonstrate the deployment of CalmBelt in a hospital diagnostic laboratory, where ONT minION (10) was used to generate SARS-CoV-2 whole-genome sequences from clinical samples on-site. CalmBelt enabled actionable information to be generated within 12 h following the detection of SARS-CoV-2 in a clinical sample. This ability to rapidly sequence and analyse SARS-CoV-2 whole-genome data enabled the swift implementation of local infection control measures, which are particularly important in suspected healthcare-associated transmission clusters.

## Materials and Methods

### On-Site SARS-CoV-2 Genome Sequencing

Nasopharyngeal swab samples were routinely tested using the Cepheid Xpert Xpress SARS-CoV-2 assay or the Roche cobas SARS-CoV-2 test. The routine diagnostic tests were performed in a CAP-accredited laboratory in a tertiary hospital, in biosafety level 2 plus containment. In brief, laboratory personnel in full PPE (18) performed all sample processing and chemical inactivation within a biosafety cabinet. All nasopharyngeal swabs were chemically inactivated (19, 20) for 30 min prior to transfer to the GeneXpert Infinity (Cepheid) in biosafety level 2 containment, or cobas 6800 System (Roche) in biosafety level 2 plus containment, for the SARS-CoV-2 tests. Remnant clinical samples found to be positive for SARS-CoV-2 RNA, with a Ct value of <31 were subjected to an additional round of chemical inactivation (19, 20) within the biosafety cabinet within biosafety level 2 plus containment, in the same laboratory. Total nucleic acid extraction of the chemically inactivated remnant samples were performed using the NucliSENS easyMag (bioMerieux) platform. The resultant total nucleic acid extract was used for downstream RT-PCR, and sequencing on ONT MinION in accordance with the ARTIC protocol v3 (21), within the same laboratory, in biosafety level 2 containment. A maximum of 25 clinical samples was sequenced on an ONT MinION sequencer in a single run. The laboratory has the capability to obtain SARS-CoV-2 sequence data from 50 clinical samples with two available ONT MinION setups. RAMPART protocol (22) was used to monitor the depth of coverage for each sample and to construct a draft genome. A previously sequenced clinical sample and a negative control (no template control) were included as positive and negative controls in every run.

### SARS-CoV-2 Genome Data

SARS-CoV-2 genomes collected in Singapore were obtained from GISAID (3,406 genomes as of 30 June 2021; Supplementary File 1). Genomes data collected from non-human hosts and environment were discarded. Missing collection dates were imputed with the first date of the respective month. Clade and lineage information were also downloaded from GISAID, where the clade the trends were calculated based on rolling average (7 days). Specific information of 99 de-identified positive cases used in the case studies was collected in Singapore General Hospital between January to August 2020 (Supplementary File 2).

### Identifying Mutation

Each SARS-CoV-2 genome obtained from on-site sequencing or GISAID was aligned to the standard SARS-CoV-2 reference genome (NC_045512.2) (23), using blastn. The alignment output in XML format was used for identifying single nucleotide polymorphisms (SNPs), insertion and deletion (Indels) with respect to the nucleotide position of the reference genome. For the genomes with multiple alignment blocks, the priority for identifying SNPs and Indels in the overlapping region was given to the longer alignment block.

### Visualization of Overall Trends

To visualize the overall trends within a country, we employ two different types; a 2D scatter plot via tSNE (24) and an interactive dendrogram. We begin by calculating nucleotide diversity at each position using


$$\begin{array}{l} Diversity\;=-\underset{i\in \left\{A,T,C,G\right\}}{\sum }x_{i}log\left(x_{i}\right) \end{array}$$

where *x*_*i*_ is a frequency of each nucleotide and $\underset{i\in \left\{A,T,C,G\right\}}{\sum }x_{i}=\;1$.

We then choose top 50 positions with highest diversity and positions that are predefined in GISAID (25). The high diversity positions were then used for calculating hamming distance among all 3,406 genomes for tSNE and dendrogram plots. For tSNE plot, we also applied k-means clustering, where k was set to 8, which is equal to the number lineages predefined by WHO.

### Outbreak Tracking Using Whole-Genome Information

For outbreak tracking, users can input multiple SARS-CoV-2 genomes (the maximum number of input genomes ≤200) obtained from the on-site sequencing platform. The input file should be in fasta format, where each input file contains one genome. The file name must follow *SampleID_dd-mm-yyyy_GroupName.fasta*, which consists of sampling identifier, collection date and predefined group name such as a location.

Each input genome is aligned to the SARS-CoV-2 reference genome using blastn, and GISAID clade is reported (25) (Supplementary File 3). A standard lineage identification model (version 3.1.15) was installed for predicting PANGO lineage of each input genome (11), in line with the nomenclature used worldwide. CalmBelt also alerts users when the breadth of coverage is <95%, allowing users to carefully analyse low-quality genome data.

To construct a phylogenetic tree of the input genomes, we followed the steps used for determining PANGO lineage by using these specific options “*mafft –retree 2 concat.fasta* > *concat.mafft”* and “*iqtree2 -s concat.mafft -m GTR*+*G”* (11). Similar to Nextstrain, we applied TreeTime (26) to generate time stamped phylogenies (Nextstrain's transmission tree) of the input genomes. To generate a similarity tree of the input genomes, whole-genome alignment information from blastn was used for calculating normalized hamming distance, and UPGMA clustering was applied on the distance matrix. In addition, as the hierarchical clustering process could introduce ambiguity for a large number of closely related genomes, a heatmap of distance matrix was also displayed to provide a quantitative measure of relatedness (i.e., number of different nucleotide positions).

An interactive similarity tree is drawn based on the whole-genome distance matrix alongside the collection date. Each node is colored according to the group name. Within the interactive tree, CalmBelt reports a subset of mutations and amino acid changes (with respect to blastn result). In the case that there are <3 nucleotides (i.e., frameshift mutations) to translate for an amino acid, the amino acid is marked as “X.” A full list of mutations is reported in a downloadable table. Monthly statistics of each PANGO lineage or sublineage in each country is also provided.

### Inspecting Changes Within the Genomic Region of Interest

We implemented a portal where users can inspect specific genomic regions and mutations of interest of the genomes (3,406 genomes from GISAID) that have been processed in the previous step. Once users input the region of interest, SNPs and Indels are reported in multiple interactive charts. The charts display both nucleotide and amino acid changes that occurred in each month. To inspect concurrent mutations or variants of concerns, the portal reports the number of occurrences based on a list of mutations from users. These features are useful for detecting diagnostic-escape by inspecting primer binding sites and inspecting concurrent mutations.

### Software and Packages

For implementing CalmBelt and analyses, we used multiple Python packages including pandas 1.1.3, matplotlib 3.3.2, numpy 1.19.2, scipy 1.5.2, seaborn 0.11.0, plotly 4.14.3, ray 1.1.0, sklearn 0.23.2, biopython 1.78, dash 1.19.0. Additional software packages include Blastn 2.6 (15), Pango 3.1.7 (11), Mafft (–retree 2) (16), IQTREE-2 (-m GTR+G) (17), and TreeTime (26). CalmBelt's source code and an installation guideline are available at https://github.com/BioML-CM/CalmBelt. The demo version and example data is available at https://calmbelt-demo.mtms.dev.

## Results

### Whole Genome Characterization and Clustering for Outbreak Investigation

**Figure 1:**
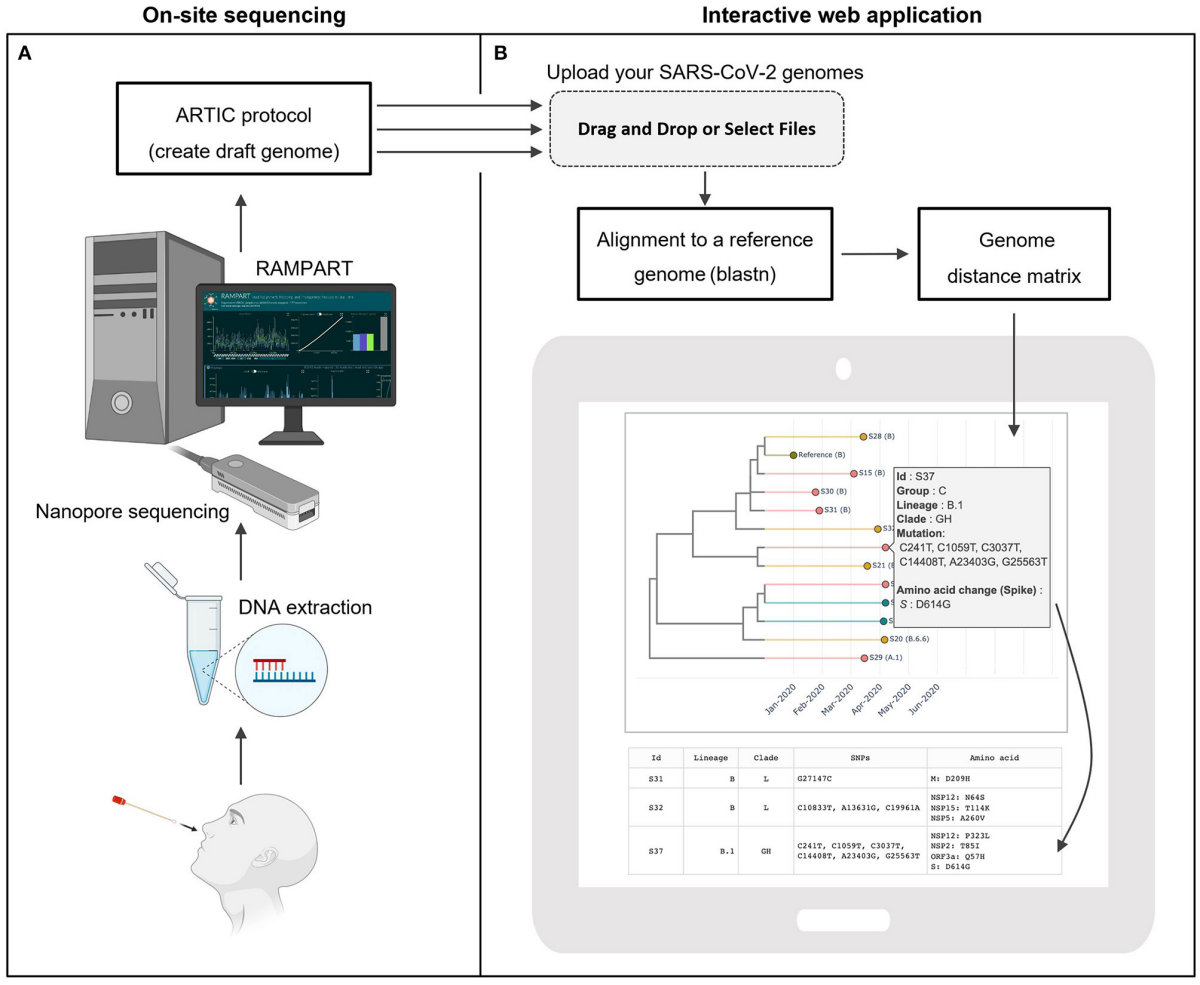
CalmBelt overview. **(A)** On-site sequencing protocol based on the Nanopore sequencing platform and ARCTIC protocol for sequencing and generating draft genomes. **(B)** Rapid analysis pipeline. Multiple SARS-CoV-2 genomes are aligned to the reference genome. A distance matrix calculated based on whole-genome alignment is used for hierarchical clustering. CalmBelt automatically generates an interactive tree capturing whole genome similarity, collection date (x-axis) and case information (color).

Nasopharyngeal swab samples were routinely tested using the Cepheid Xpert Xpress SARS-CoV-2 assay or the Roche cobas SARS-CoV-2 test. Samples found to be positive for SARS-CoV-2 RNA, with a Ct value of <31 were subjected to total nucleic acid extraction using the NucliSENS easyMag (bioMerieux) platform. The total nucleic acid extract was used for downstream RT-PCR, and sequencing on ONT MinION in accordance with the ARTIC protocol v3 (21). RAMPART protocol (22) was used to monitor the depth of coverage for each sample and to construct a draft genome (**Methods**; Figure 1A).

Once the draft genome became available, Users dragged and dropped the SARS-CoV-2 genome files into the analysis interface (Figure 1B). Multiple genomes from on-site sequencing were aligned to the reference genome. ClamBelt then identified mutations, calculated whole genome similarity, and constructed multiple trees for transmission tracking (**Methods**). As the number of mutations per genome continued to increase (~2–3 mutations per month; Supplementary Figure 1), CalmBelt only visualized a subset of mutations while providing the downloadable full list of mutations.

The similarity tree captures hierarchical clustering results based on the whole genome similarity. The x-axis represents collection date, and colors represent case-specific information such as locations, as defined by the users. CalmBelt integrates existing nomenclatures including GISAID clade (9, 25) and PANGO lineages (12, 27). For each case, comprehensive information including mutations at nucleotide- and amino acid-level can be displayed interactively.

As highlighted in the Nextstrain web portal (28), “copying mistakes” that accumulate in the genome could facilitate tracking of transmission routes and dynamics, while a phylogenetic tree could infer locations of each case. CalmBelt therefore visualizes multiple types of trees, as well as a distance matrix capturing pairwise distance between SARS-CoV-2 genomes, to provide multiple perspectives for epidemiology units (Figure 1B; Supplementary Figure 2).

**Figure 2:**
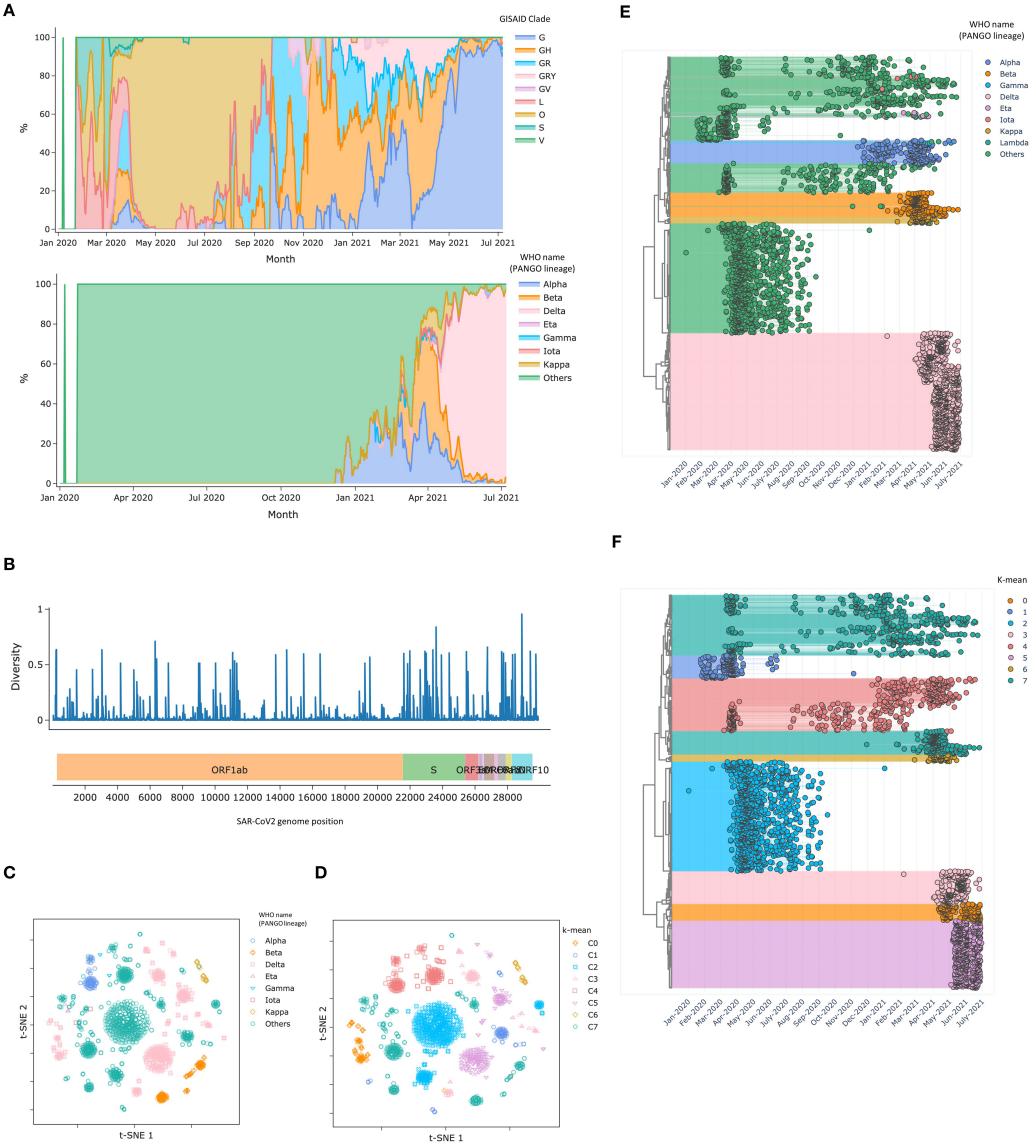
Statistics of SARS-CoV-2 genomes in Singapore (January 2020–June 2021). **(A)** A time series presenting proportion of different clades based on GISAID criteria and the newly defined lineage name according to WHO. **(B)** Nucleotide diversity across SARS-CoV-2 genomic loci **(C,D)** T-SNE plots visualize clusters of SAR-CoV2 genomes, where the distances are based on high diversity loci **(E,F)** Dendrograms present different SAR-CoV2 genome along with collection date and lineage information.

Besides, CalmBelt analyzed overall statistics for a large set of 3,406 genomes from Singapore (Figure 2, Methods). Users can inspect the statistics based on genomic information in terms of GISAID clade, which captures the trends at the beginning of the pandemic, and the newly defined PANGO lineage name according to WHO (Figure 2A). For example, we observed a clear dominant clade from April 2020 to September 2020, corresponding to the foreign worker dormitory outbreak in Singapore (29). Apart from the period of dormitory outbreak, the trends we observed in the Singapore data are more similar to the trend observed across countries in Asia compared to the trends observed in other continents, in line with geographical information (Supplementary Figures 3, 4).

Similar to Nextstrain (8), nucleotide diversity was calculated for each genome position. We observed various mutation rates across different genes (Figure 2B). While mutations in the S gene, which encodes the Spike protein (30) is of particular interest due to its role in infectivity and vaccine efficacy, other genes (e.g., E gene, N gene) are monitored as they are commonly used as diagnostic targets (31). We note that CalmBelt shows information from all genomes without subsampling, a pre-processing step used in Nextstrain.

Genomic loci with high diversity were used for visualization (Figures 2C–F). Overlaying PANGO lineage information onto the 2D visualization, we observed multiple clusters that have the same lineage name (Figures 2C,E). We found that clustering based on high diversity genomic loci could capture the foreign worker dormitory outbreak (C2; Figures 2D,F) and distinguished Delta (B.1.617.2) into three different clusters (C0, C3, C5) that spreaded at distinct time periods, suggesting different sources of the outbreak even though all cases were annotated as Delta based on the newly assigned PANGO lineage.

### Investigating Healthcare-Associated SARS-CoV-2 Transmission

**Figure 3:**
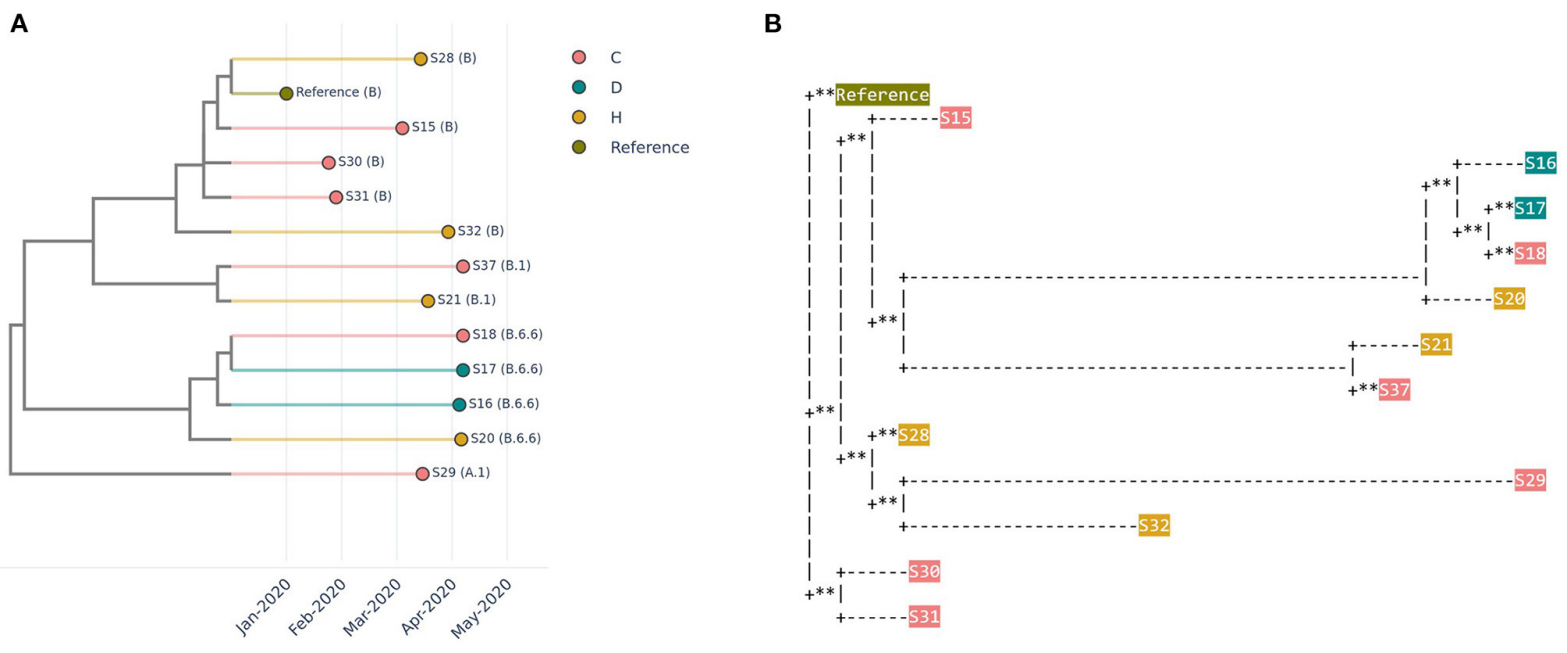
Relationship of 12 SARS-CoV-2 genomes observed in the community (C), healthcare workers (H), and dormitory residents (D). **(A)** A similarity tree is based on a combination of whole genome similarity and collection date. **(B)** A phylogenetic tree based on multiple sequence alignment and a predefined mutation rate model (Mafft and IQ-TREE2), where ^**^ indicate zero distance.

Four healthcare workers working in the same institution were found to be positive for SARS-CoV-2 within a short span of time. There were concerns of healthcare-associated/occupationally acquired SARS-CoV-2 infections among the healthcare workers. Therefore, on-site sequencing was performed for 4 healthcare workers' samples, along with 6 samples from the community and 2 samples from dormitory residents. A similar pattern was observed across different types of trees (Figures 3A,B, Methods). The similarity and transmission trees had the added advantage of capturing temporal relationships based on collection date information, which are useful adjuncts in outbreak investigations. Based on CalmBeIt's outputs, it was concluded that sequences from the four de-identified healthcare workers belonged to different clusters, suggesting that SARS-CoV-2 transmission did not occur in the healthcare or occupational setting in this instance.

To enhance the ability to identify potential sources of epidemiologically unlinked cases, CalmBelt allows users to query for statistics of a given PANGO lineage in different countries for a specific month. For example, in March 2020, B.6.6 was widespread in Cambodia (2/19, 10.53%), Singapore (36/381, 9.45%) and Malaysia (5/56, 8.93%) (Supplementary Figure 5). This information will allow users to identify potential links for further epidemiological investigations.

### Mining Transmission Patterns in a Large Outbreak

**Figure 4:**
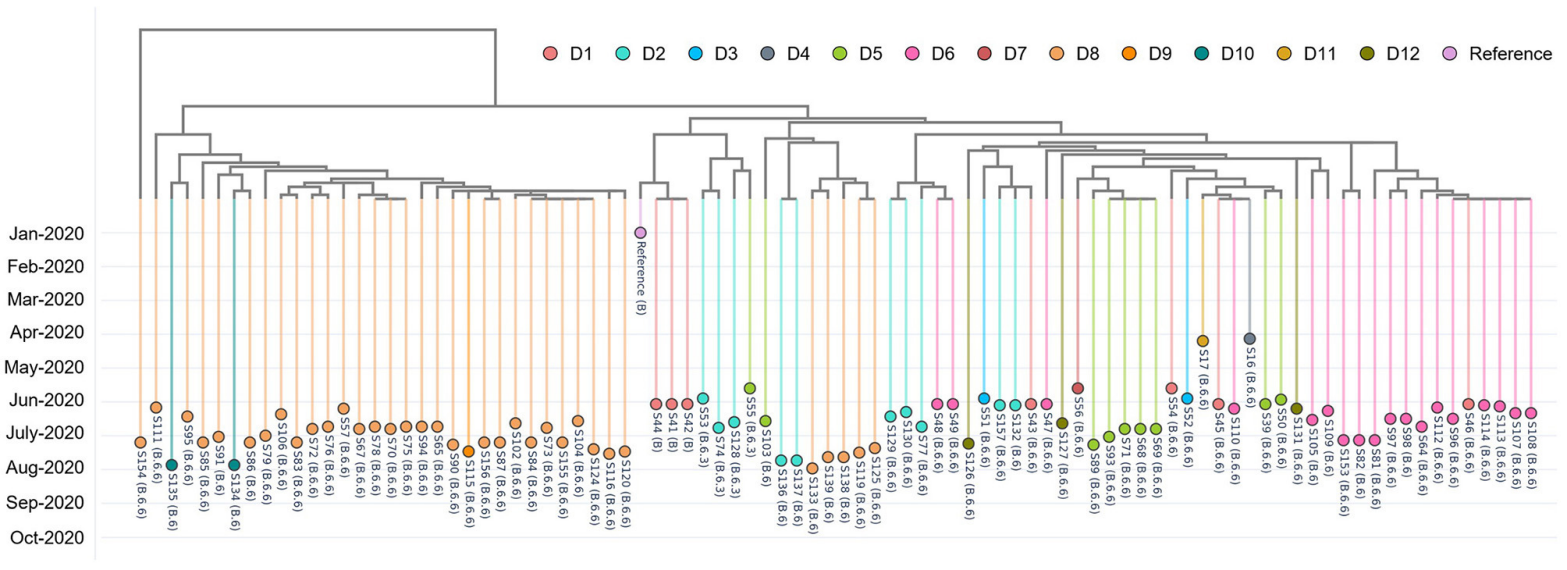
A genome similarity tree shows the relationship among 89 randomly selected, de-identified positive cases which were labeled to be from dormitory residents. These positive cases were detected from 12 dormitories (D1–D12) from the foreign worker dormitory outbreak in Singapore (April–August 2020). Each data point represents a positive case, and colors represent different, de-identified dormitories.

We inspected 89 randomly selected, de-identified positive cases from the foreign worker dormitory outbreak in Singapore between April to August 2020. Using CalmBelt's interactive tree, we observed two main clusters (Figure 4). In the first cluster (orange color; left), the majority of the cases were from a single dormitory (D8), two cases were from D10 and one case was from D9. However, there are 5 cases from D8 in the second cluster, suggesting that there were at least two sources of the infection in D8. In the second cluster (mixed colors; right), cases from many dormitories were observed. Cases from the same dormitory tend to form a cluster, highlighting the spread of the SARS-CoV-2 virus within each dormitory. In addition, we observed multiple small clusters in some dormitories (e.g., within D2, S53 + S74 + S128, S136 + S137, S129 + S130 + S77, S157 + S132), which may suggest multiple sources and routes of transmission within D2.

While PANGO lineages are meant to delineate outbreaks, in the setting of a rapidly disseminating local outbreaks, the resolution offered by PANGO lineage assignment alone is likely to be insufficient. For example, in the case of this large-scale dormitory outbreak, the majority of the cases (73%) were annotated as B.6.6. CalmBelt was able to capture whole genome similarity and increased the resolution, thereby delineating possible clusters within each dormitory.

### Monitoring Possible Diagnostic-Escape

**Figure 5:**
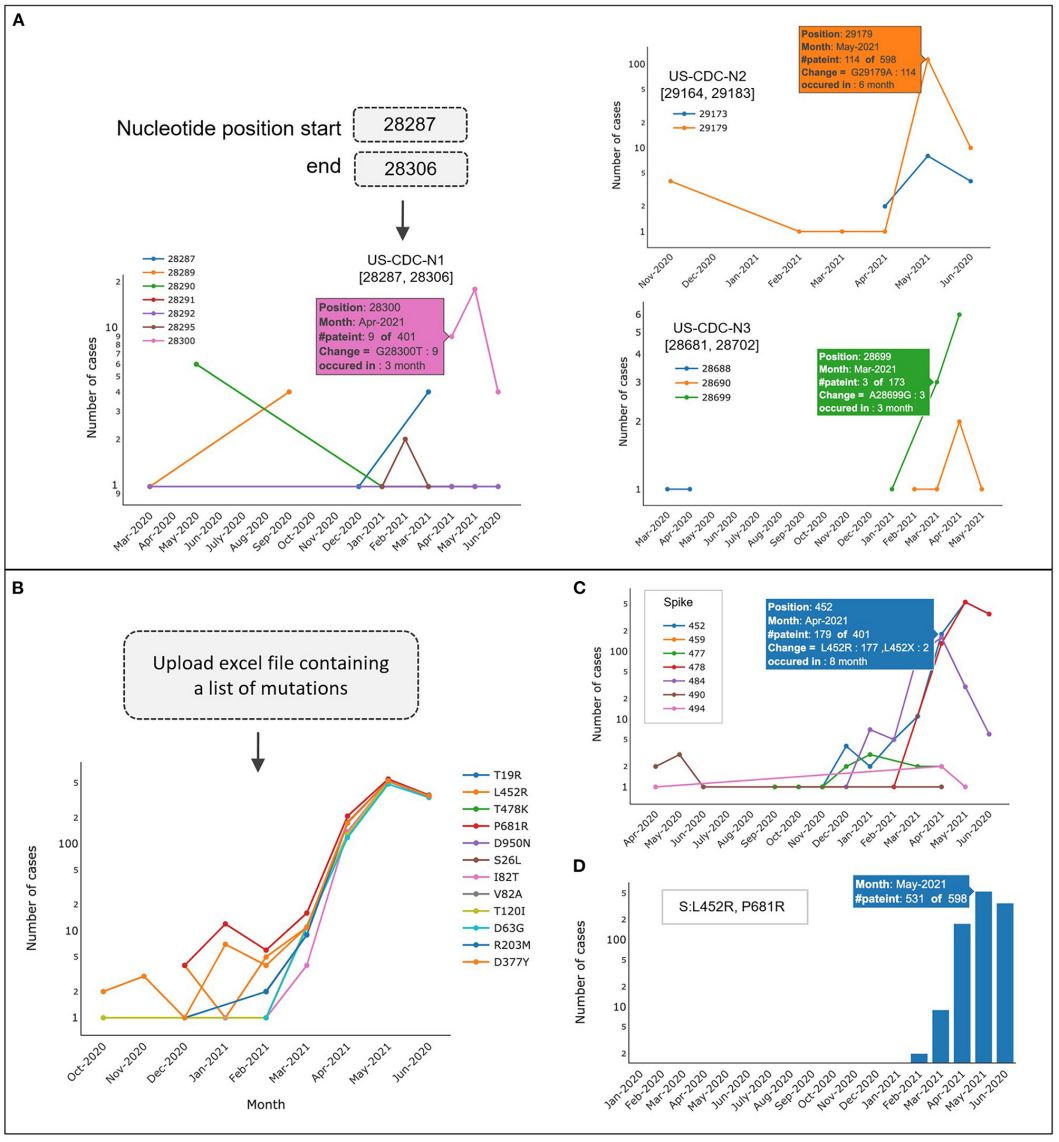
Inspecting mutations in a specific region for each month. **(A)** Mutation frequencies of three genomic regions in the N gene (PCR primers US-CDC-N1 to N3). **(B–D)** CalmBelt reports the number of cases for each amino acid change (missense mutation) according to user input. **(D)** Number of cases harboring co-occurrence amino acid changes (L452R and P681R in S gene).

CalmBelt can be used to monitor genomic mutations and allow users to investigate genomic regions of interest. As a use case, we selected three genomic regions in the N gene, based on PCR primers used for the detection of N gene [US-CDC-N1 to N3 (32); Figure 5A]. We found that mutations occurred in <5% of Singapore sequences (18 out of 598 and 6 out of 401) in the regions primed by US-CDC-N1 and US-CDC-N3. However, for the region primed by US-CDC-N2, we observed a higher percentage (19%) of Singapore sequences that harbor a mutation at position 29,179. This tool therefore allows users to monitor for emerging diagnostic escapes, based on the local laboratories' primer sequences and relevant regional SARS-CoV-2 sequence data.

As the number of mutations per genome continued to accumulate, ~2–3 new mutations emerged per month (Supplementary Figure 1). CalmBelt allows users to input a custom list of mutations and reports the number of cases where each mutation is present. For example, we added a list of mutations occurring in B.1.617.2 lineage or Delta (13) including variants of biological significance S:P681R and S:L452R which are associate with viral infectivity and reduction in antibody neutralization in the presence of other mutations in S gene (33, 34) (Figure 5B). Alternatively, users can input a protein name and a range of amino acid loci (Figure 5C). CalmBelt also allows users to input a specific set of mutations to observe the co-occurring frequency in each month (Figure 5D). In our example, we found that the co-occurrence of two variants (S:P681R and S:L452R) is well-preserved, which suggests biological significance that warrants further investigations (35).

## Discussion

We present CalmBelt, a streamlined framework for on-site sequencing and analysis of SARS-CoV-2 genomes for outbreak tracking (Figures 1, 2). Using the nanopore sequencing platform, we initiated sequencing on-site within the hospital diagnostic laboratory once clinical samples were found to be positive by routine RT-PCR testing. While nanopore technologies have significantly improved accessibility to sequencing capability, the ability to analyze sequence data within an actionable time frame remains a challenge for many hospital laboratories. CalmBelt was developed to close this gap by allowing typical diagnostic laboratory users with no formal bioinformatic training to analyze whole genome sequence data to facilitate outbreak investigations and control in the healthcare settings.

When CalmBelt was fully incorporated into the diagnostic laboratory's workflow, <24 h was required from nasopharyngeal swab sample receipt to CalmBelt outputs. This was achieved by the use of a routine RT-PCR diagnostic test with rapid turnaround time (Cepheid Xpert Xpress SARS-CoV-2 assay), as well as a workflow that initiated downstream processing for nanopore sequencing as soon as the sample was found to be positive for SARS-CoV-2. Calmbelt could generate an online report capturing relatedness of the samples analyzed, within 24 h of sample arrival in the laboratory, which is a timeframe compatible with infection control mitigations in the healthcare setting (Figures 3, 4).

The repeated, rapid emergence of SARS-CoV-2 variants necessitates constant vigilance against variants that may escape routine diagnostic detection by commonly used RT-PCR assays (31, 36). To that end, CalmBelt was designed to help users monitor for mutations in primer-binding sites, which is an important safeguard against plausible diagnostic-escapes (Figure 5). The curated genomes in the GISAID SARS-CoV-2 database provides a great resource for us to gather mutation information within a specific country and across the globe, allowing us to track mutation patterns in certain genomic regions to monitor plausible diagnostic-escape (31). Even when proprietary primer sequences are used in commercial RT-PCR assays, CalmBelt allows mutations in the targeted gene(s) to be monitored by the users, and questions can be raised to assay manufacturers if any unusual trends are observed.

A major criticism of the nanopore platform has been the suboptimal sequencing accuracy. Using the improved ARCTIC protocol (21), we were able to obtain high accuracy genomes from most of the samples. Various recent studies similarly reported the successful use of nanopore platforms to obtain SARS-CoV-2 whole-genome sequences (10, 37, 38), and thousands of SARS-CoV-2 genomes submitted to GISAID were derived solely from nanopore sequencing. This demonstrates that the nanopore platform is able to generate adequately accurate SARS-CoV-2 sequence data for real-time downstream analyses. Given the small footprint, relatively low setup cost, nanopore will continue to play an important role in democratizing access to sequencing.

The B.1.617.2 (Delta) variant has rapidly become the dominant variant in Singapore by mid 2021. To delineate outbreak clusters among cases infected with B.1.617.2, CalmBelt was designed to achieve a higher resolution than the widely used PANGO lineage. Besides the suboptimal performance of the machine learning model for PANGO lineage prediction, as the number of accumulated mutations in SARS-CoV-2 genomes is growing (39, 40), a single large phylogenetic tree could lose the resolution of retrospective cases (Figure 4) as well as recent cases, where most cases are identified as Delta despite coming from different community clusters (8).

Several web applications for SARS-CoV-2 sequence analyses, such as Nextstrain (9), Audacity, PrimerChecker (9) and CoVariants (41), have been recently developed. However, none of these tools (except Nextstrain) focus on integrating custom user input and sample collection dates to create a specialized report for outbreak investigations and control mitigations in the healthcare settings. With the availability of nanopore sequencing technologies, sentinel clinical diagnostic laboratories performing routine SARS-CoV-2 RT-PCR testing are in the position to generate SARS-CoV-2 sequence data most rapidly and seamlessly from clinical samples and contribute to rapid local outbreak response. By integrating rapid diagnostic testing, on-site nanopore sequencing and intuitive genome analysis afforded by CalmBelt, we were able to generate outbreak reports from clinical samples within a day. In the event of sensitive clusters, such as hospitals, senior care homes and schools, the quick availability of such reports will complement manual contact tracing and contribute to faster and better control of spread among vulnerable or unvaccinated populations.

Although the use of the nanopore platform and easy-to-use informatic tools such as CalmBelt have made SARS-CoV-2 sequencing and analysis increasingly accessible, hurdles remain for large scale implementation. The consumable and manpower cost continue to be a challenge in resource-limited settings, as clinical diagnostic laboratories are generally designed and budgeted based on large-volume routine testing. While the COVID-19 pandemic might have accelerated the capital investment and development of molecular diagnostics in routine clinical laboratories, manpower well-versed in the sequence data interpretation and application may constrain implementation. Therefore, further development and human capital and sequencing laboratory setups are necessary to encourage large scale implementation of SARS-CoV-2 sequencing.

While the data presented in this paper is tailored to SARS-CoV-2 genome analysis, our bioinformatic workflow can be easily adapted for other viruses where the sequencing workflow can follow ARTIC network protocols (21). This versatility allows users deploying CalmBelt to build preparedness and resilience for outbreaks and pandemics beyond the current COVID-19 pandemic. Our strategy also demonstrated the feasibility of a rapid whole-genome sequencing framework in the healthcare setting, paving the way for further applications of rapid sequencing technologies in the clinical diagnostics.

## Data Availability Statement

The original contributions presented in the study are included in the article/Supplementary Material, further inquiries can be directed to the corresponding author/s.

## Author Contributions

HY, KK, and CS planned and designed the project. HY developed a CalmBelt web application and performed all computational analysis with CS's supervision. NA and ST planned and performed wet-lab experiments with KK's supervision. NN provides feedback on computational and wet-lab experiments, as well as manuscripts. HY, KK, and CS wrote the manuscript with input from all authors. All authors contributed to the article and approved the submitted version.

## Funding

This work was supported by funding from Chiang Mai University, SingHealth Duke-NUS Academic Medicine [Grant Number AM/CSP012/2020 (SRDUKAMC2012)] and A^*^STAR. KK was supported by the Singapore National Medical Research Council research training fellowship.

## Conflict of Interest

The authors declare that the research was conducted in the absence of any commercial or financial relationships that could be construed as a potential conflict of interest.

## Publisher's Note

All claims expressed in this article are solely those of the authors and do not necessarily represent those of their affiliated organizations, or those of the publisher, the editors and the reviewers. Any product that may be evaluated in this article, or claim that may be made by its manufacturer, is not guaranteed or endorsed by the publisher.

## Supplementary Material

Supplementary File 1 Acknowledgment table SARS-CoV-2 genomes collected in Singapore from GISAID (30 June 2021).

Supplementary File 2 Detail of 99 de-identified positive cases collected in Singapore General Hospital (January to August 2020).

Supplementary File 3 Mutations for GISAID clades.

Supplementary Figure 1 Increasing mutation rate of 3,406 genomes from Singapore downloaded from GISAID (Jan 2020–June 2021). Y-axis represents an average number of mutations per week.

Supplementary Figure 2 In addition to a similarity tree and a list of mutations in Figure 1B, CalmBelt provides a phylogenetic tree (**A**; IQTREE2 used in Pangolin), a transmission tree (**B**; TreeTime used in Nextstrain), and a heatmap **(C)** capturing the number of different nucleotides between every pair of genomes.

Supplementary Figure 3 Time-series plots show the trend of GISAID clades across continents. We note that the trends might be slightly different from Nextstrain due to the subsampling process used in Nextstrain.

Supplementary Figure 4 Time-series plots show the trend of PANGO lineage (based on the greek alphabets) across continents.

Supplementary Figure 5 Statistics of B.6.6 lineage across different countries between March to May 2020.
