# Supplementary material for: CalmBelt: Rapid SARS-CoV-2 Genome Characterization for Outbreak Tracking
Source: Front Med (Lausanne). 2021 Dec 14;8:790662. doi: 10.3389/fmed.2021.790662 (PMC8712659; doi:10.3389/fmed.2021.790662)
Supplement: Supplementary File 3 — Mutations for GISAID clades. [file Data_Sheet_2.PDF]

We gratefully acknowledge the following Authors from the Originating laboratories responsible for obtaining the specimens, as well as the Submitting laboratories where the genome data were generated and shared via GISAID, on which this research is based.

All Submitters of data may be contacted directly via www.gisaid.org

Authors are sorted alphabetically.

Accession ID	Originating Laboratory	Submitting Laboratory	Authors
EPI_ISL_1034260, EPI_ISL_1034261, EPI_ISL_1034262, EPI_ISL_1034263, EPI_ISL_1034264, EPI_ISL_1034265, EPI_ISL_1034266, EPI_ISL_1034267, EPI_ISL_1034268, EPI_ISL_1034269, EPI_ISL_1034270, EPI_ISL_1034271, EPI_ISL_1034272, EPI_ISL_1034273, EPI_ISL_1081922, EPI_ISL_1081924, EPI_ISL_1081926, EPI_ISL_1081928, EPI_ISL_1081930, EPI_ISL_1081932, EPI_ISL_1081934, EPI_ISL_1081935, EPI_ISL_1081937, EPI_ISL_1081939, EPI_ISL_1081941, EPI_ISL_1081943, EPI_ISL_1081945, EPI_ISL_1081946, EPI_ISL_1081947, EPI_ISL_1081948, EPI_ISL_1081949, EPI_ISL_1081950, EPI_ISL_1081951, EPI_ISL_1081952, EPI_ISL_1098833, EPI_ISL_1098834, EPI_ISL_1098835, EPI_ISL_1098836, EPI_ISL_1098837, EPI_ISL_1164353, EPI_ISL_1164354, EPI_ISL_1164355, EPI_ISL_1164356, EPI_ISL_1164357, EPI_ISL_1164358, EPI_ISL_1164359			
see above	National Public Health Laboratory, National Centre for Infectious Diseases	National Public Health Laboratory, National Centre for Infectious Diseases	Tze Minn Mak, Zhenyang Zhou, Lin Cui, Raymond Tzer Pin Lin
EPI_ISL_1173248, EPI_ISL_1173249, EPI_ISL_1173250, EPI_ISL_1173251, EPI_ISL_1173252, EPI_ISL_1173253, EPI_ISL_1173254, EPI_ISL_1173255, EPI_ISL_1173256	National Public Health Laboratory, National Centre for Infectious Diseases	National Public Health Laboratory, National Centre for Infectious Diseases	Tze Minn Mak, Zhenyang Zhou, Royce Ang, Lin Cui, Raymond Tzer Pin Lin
EPI_ISL_1229162, EPI_ISL_1229163, EPI_ISL_1229164, EPI_ISL_1229165, EPI_ISL_1229166, EPI_ISL_1229167, EPI_ISL_1229168, EPI_ISL_1252446, EPI_ISL_1252447, EPI_ISL_1252448, EPI_ISL_1252449, EPI_ISL_1252450, EPI_ISL_1252451, EPI_ISL_1252452, EPI_ISL_1252453, EPI_ISL_1252454, EPI_ISL_1252455, EPI_ISL_1295938, EPI_ISL_1295939, EPI_ISL_1295940, EPI_ISL_1295941, EPI_ISL_1295942, EPI_ISL_1312382, EPI_ISL_1312383, EPI_ISL_1312384, EPI_ISL_1312385, EPI_ISL_1312386, EPI_ISL_1312387, EPI_ISL_1312388			
see above	National Public Health Laboratory, National Centre for Infectious Diseases	National Public Health Laboratory, National Centre for Infectious Diseases	Tze Minn Mak, Zhenyang Zhou, Grace Jie Yin Ngan, Royce Ang, Lin Cui, Raymond Tzer Pin Lin
EPI_ISL_1315626, EPI_ISL_1315627	National Public Health Laboratory, National Centre for Infectious Diseases	National Public Health Laboratory, National Centre for Infectious Diseases	Tze Minn Mak, Zhenyang Zhou, Lin Cui, Raymond Tzer Pin Lin
EPI_ISL_1315628, EPI_ISL_1367545, EPI_ISL_1367546, EPI_ISL_1367547, EPI_ISL_1367548, EPI_ISL_1367549, EPI_ISL_1367550, EPI_ISL_1367551, EPI_ISL_1367552, EPI_ISL_1367553, EPI_ISL_1367554, EPI_ISL_1367555, EPI_ISL_1367556, EPI_ISL_1367557, EPI_ISL_1367558, EPI_ISL_1367559, EPI_ISL_1367560, EPI_ISL_1367561, EPI_ISL_1367562, EPI_ISL_1367563, EPI_ISL_1367564, EPI_ISL_1367565, EPI_ISL_1442942, EPI_ISL_1442943, EPI_ISL_1442944, EPI_ISL_1442945, EPI_ISL_1442946, EPI_ISL_1442947, EPI_ISL_1442948, EPI_ISL_1442949, EPI_ISL_1442950, EPI_ISL_1442951, EPI_ISL_1442952, EPI_ISL_1442953, EPI_ISL_1442954, EPI_ISL_1442955, EPI_ISL_1476987, EPI_ISL_1476988, EPI_ISL_1476989, EPI_ISL_1476990, EPI_ISL_1476991, EPI_ISL_1476992, EPI_ISL_1476993, EPI_ISL_1476994, EPI_ISL_1476995, EPI_ISL_1476996, EPI_ISL_1476997, EPI_ISL_1476998, EPI_ISL_1476999, EPI_ISL_1477000, EPI_ISL_1477001, EPI_ISL_1477002, EPI_ISL_1477003, EPI_ISL_1477004, EPI_ISL_1477005, EPI_ISL_1477006, EPI_ISL_1477007, EPI_ISL_1477008, EPI_ISL_1477009, EPI_ISL_1477010, EPI_ISL_1477011, EPI_ISL_1477012, EPI_ISL_1477013, EPI_ISL_1477014, EPI_ISL_1477015, EPI_ISL_1477016, EPI_ISL_1477017, EPI_ISL_1477018, EPI_ISL_1477019, EPI_ISL_1477020, EPI_ISL_1477021, EPI_ISL_1477022, EPI_ISL_1477023, EPI_ISL_1477024, EPI_ISL_1477025, EPI_ISL_1477026, EPI_ISL_1477027, EPI_ISL_1477028, EPI_ISL_1477029, EPI_ISL_1477030, EPI_ISL_1477031, EPI_ISL_1477032, EPI_ISL_1477033, EPI_ISL_1477034, EPI_ISL_1477035, EPI_ISL_1477036, EPI_ISL_1477037, EPI_ISL_1477038, EPI_ISL_1477039, EPI_ISL_1477040, EPI_ISL_1477041, EPI_ISL_1477042, EPI_ISL_1489717, EPI_ISL_1489718, EPI_ISL_1489719, EPI_ISL_1489720, EPI_ISL_1489721, EPI_ISL_1489722, EPI_ISL_1489723, EPI_ISL_1489724, EPI_ISL_1489725, EPI_ISL_1489726, EPI_ISL_1489727, EPI_ISL_1489728, EPI_ISL_1519355, EPI_ISL_1519356, EPI_ISL_1519357, EPI_ISL_1519358, EPI_ISL_1519360, EPI_ISL_1519361, EPI_ISL_1519362, EPI_ISL_1519363, EPI_ISL_1519364, EPI_ISL_1519365, EPI_ISL_1519366, EPI_ISL_1519368, EPI_ISL_1519369, EPI_ISL_1519370, EPI_ISL_1519371, EPI_ISL_1519372, EPI_ISL_1519373, EPI_ISL_1519374, EPI_ISL_1519375, EPI_ISL_1519377, EPI_ISL_1519378, EPI_ISL_1519379, EPI_ISL_1519380, EPI_ISL_1519381, EPI_ISL_1519382, EPI_ISL_1519383, EPI_ISL_1519384, EPI_ISL_1519386, EPI_ISL_1519388, EPI_ISL_1519391, EPI_ISL_1519393, EPI_ISL_1519396, EPI_ISL_1519398, EPI_ISL_1519401, EPI_ISL_1519405, EPI_ISL_1519407, EPI_ISL_1519410, EPI_ISL_1519413, EPI_ISL_1519416, EPI_ISL_1519419, EPI_ISL_1524777, EPI_ISL_1524778, EPI_ISL_1524779, EPI_ISL_1524780, EPI_ISL_1524781, EPI_ISL_1524782, EPI_ISL_1524783, EPI_ISL_1524784, EPI_ISL_1524785, EPI_ISL_1524786, EPI_ISL_1524787, EPI_ISL_1524788, EPI_ISL_1524789, EPI_ISL_1524790, EPI_ISL_1524791, EPI_ISL_1524792, EPI_ISL_1524793, EPI_ISL_1524794, EPI_ISL_1524795, EPI_ISL_1524796, EPI_ISL_1524797, EPI_ISL_1524798, EPI_ISL_1524799, EPI_ISL_1524800, EPI_ISL_1524801, EPI_ISL_1543939, EPI_ISL_1543940, EPI_ISL_1543941, EPI_ISL_1543942, EPI_ISL_1543943, EPI_ISL_1543944, EPI_ISL_1543945, EPI_ISL_1543946, EPI_ISL_1543947, EPI_ISL_1543948, EPI_ISL_1543949, EPI_ISL_1543950, EPI_ISL_1543951, EPI_ISL_1543952, EPI_ISL_1543953, EPI_ISL_1543954, EPI_ISL_1543955, EPI_ISL_1543956, EPI_ISL_1543957, EPI_ISL_1543958, EPI_ISL_1543959, EPI_ISL_1543960, EPI_ISL_1543961, EPI_ISL_1543962, EPI_ISL_1543963, EPI_ISL_1543964, EPI_ISL_1543965, EPI_ISL_1543966, EPI_ISL_1543967, EPI_ISL_1543968, EPI_ISL_1543969, EPI_ISL_1543970, EPI_ISL_1543971, EPI_ISL_1543972, EPI_ISL_1543973, EPI_ISL_1543974, EPI_ISL_1543975, EPI_ISL_1543976, EPI_ISL_1543977, EPI_ISL_1543978, EPI_ISL_1543979, EPI_ISL_1543980, EPI_ISL_1543981, EPI_ISL_1543982, EPI_ISL_1545779, EPI_ISL_1568430, EPI_ISL_1568432, EPI_ISL_1620131, EPI_ISL_1620132, EPI_ISL_1620133, EPI_ISL_1620134, EPI_ISL_1620135, EPI_ISL_1620136, EPI_ISL_1620137, EPI_ISL_1620138, EPI_ISL_1620139, EPI_ISL_1620140, EPI_ISL_1620141, EPI_ISL_1620142, EPI_ISL_1620143, EPI_ISL_1620144, EPI_ISL_1620145, EPI_ISL_1620146, EPI_ISL_1620147, EPI_ISL_1620148, EPI_ISL_1620149, EPI_ISL_1620150, EPI_ISL_1620151, EPI_ISL_1620152, EPI_ISL_1620153, EPI_ISL_1620154, EPI_ISL_1620155, EPI_ISL_1620156, EPI_ISL_1620157, EPI_ISL_1620158, EPI_ISL_1620159, EPI_ISL_1620160, EPI_ISL_1620161, EPI_ISL_1620162, EPI_ISL_1620163, EPI_ISL_1620164, EPI_ISL_1620165, EPI_ISL_1620166, EPI_ISL_1620167, EPI_ISL_1620168, EPI_ISL_1620169, EPI_ISL_1620170, EPI_ISL_1634423, EPI_ISL_1634424, EPI_ISL_1634425, EPI_ISL_1634426, EPI_ISL_1634427, EPI_ISL_1634428, EPI_ISL_1634430, EPI_ISL_1634431, EPI_ISL_1634432, EPI_ISL_1634433, EPI_ISL_1652091, EPI_ISL_1652092, EPI_ISL_1652093, EPI_ISL_1652094, EPI_ISL_1652095, EPI_ISL_1652096, EPI_ISL_1652097, EPI_ISL_1652098, EPI_ISL_1652099, EPI_ISL_1652100, EPI_ISL_1652101, EPI_ISL_1652102, EPI_ISL_1652103, EPI_ISL_1652104, EPI_ISL_1652105, EPI_ISL_1652106, EPI_ISL_1652108, EPI_ISL_1652109, EPI_ISL_1652110, EPI_ISL_1652111, EPI_ISL_1652112, EPI_ISL_1652113, EPI_ISL_1652114, EPI_ISL_1652115, EPI_ISL_1652116, EPI_ISL_1652117, EPI_ISL_1652118, EPI_ISL_1652119, EPI_ISL_1652120, EPI_ISL_1652122, EPI_ISL_1652123, EPI_ISL_1652198, EPI_ISL_1652199, EPI_ISL_1652200, EPI_ISL_1652531, EPI_ISL_1652532, EPI_ISL_1668967, EPI_ISL_1704809, EPI_ISL_1704810, EPI_ISL_1704811, EPI_ISL_1704812, EPI_ISL_1704813, EPI_ISL_1704814, EPI_ISL_1704815, EPI_ISL_1704816, EPI_ISL_1704817, EPI_ISL_1704818, EPI_ISL_1704819, EPI_ISL_1704820, EPI_ISL_1704821, EPI_ISL_1704822, EPI_ISL_1704823, EPI_ISL_1704824, EPI_ISL_1704825, EPI_ISL_1704826, EPI_ISL_1704827, EPI_ISL_1704828, EPI_ISL_1704829, EPI_ISL_1704830, EPI_ISL_1704831, EPI_ISL_1704832, EPI_ISL_1704833, EPI_ISL_1704834, EPI_ISL_1704835, EPI_ISL_1704836, EPI_ISL_1704837, EPI_ISL_1704838, EPI_ISL_1704839, EPI_ISL_1704840, EPI_ISL_1704841, EPI_ISL_1704842, EPI_ISL_1704843, EPI_ISL_1704844, EPI_ISL_1704845, EPI_ISL_1704846, EPI_ISL_1704847, EPI_ISL_1704848, EPI_ISL_1704849, EPI_ISL_1704850, EPI_ISL_1704851, EPI_ISL_1719868, EPI_ISL_1719869, EPI_ISL_1719870, EPI_ISL_1719871, EPI_ISL_1719872, EPI_ISL_1719873, EPI_ISL_1719874, EPI_ISL_1719875, EPI_ISL_1719876, EPI_ISL_1719877, EPI_ISL_1719878, EPI_ISL_1719879, EPI_ISL_1719880, EPI_ISL_1719881, EPI_ISL_1719882, EPI_ISL_1719883, EPI_ISL_1719884, EPI_ISL_1719885, EPI_ISL_1719886, EPI_ISL_1719887, EPI_ISL_1719888, EPI_ISL_1719889, EPI_ISL_1719890, EPI_ISL_1719891, EPI_ISL_1719892, EPI_ISL_1719893, EPI_ISL_1719894, EPI_ISL_1719895, EPI_ISL_1719896, EPI_ISL_1719897, EPI_ISL_1719898, EPI_ISL_1719899, EPI_ISL_1719900, EPI_ISL_1719901, EPI_ISL_1719902, EPI_ISL_1719903, EPI_ISL_1719904			
see above	National Public Health Laboratory, National Centre for Infectious Diseases	National Public Health Laboratory, National Centre for Infectious Diseases	Tze Minn Mak, Zhenyang Zhou, Grace Jie Yin Ngan, Royce Ang, Lin Cui, Raymond Tzer Pin Lin
EPI_ISL_1749430, EPI_ISL_1749431, EPI_ISL_1749432, EPI_ISL_1749433, EPI_ISL_1749434, EPI_ISL_1749435, EPI_ISL_1749436, EPI_ISL_1749437	Singapore General Hospital	Department of Microbiology	Nurdyana Abdul Rahman, Kun Lee Lim, Chenhao Li, Sui Sin Goh, Kenneth Xin Long Chan, Kian Sing Chan, Lynette Oon, Kern Rei Chng, Niranjan Nagarajan, Karrie Ko
EPI_ISL_1752677, EPI_ISL_1816925, EPI_ISL_1816926, EPI_ISL_1816927, EPI_ISL_1816928, EPI_ISL_1816929, EPI_ISL_1816930, EPI_ISL_1816931, EPI_ISL_1816932, EPI_ISL_1816933, EPI_ISL_1816934, EPI_ISL_1816935, EPI_ISL_1816936, EPI_ISL_1816937, EPI_ISL_1816938, EPI_ISL_1816939, EPI_ISL_1816940, EPI_ISL_1816941, EPI_ISL_1816942, EPI_ISL_1816943, EPI_ISL_1816944, EPI_ISL_1816945, EPI_ISL_1816946, EPI_ISL_1816947, EPI_ISL_1816948, EPI_ISL_1816949, EPI_ISL_1816950, EPI_ISL_1816951, EPI_ISL_1816952, EPI_ISL_1816953, EPI_ISL_1816954, EPI_ISL_1816955, EPI_ISL_1816956, EPI_ISL_1816957, EPI_ISL_1816958, EPI_ISL_1816959, EPI_ISL_1816960, EPI_ISL_1816961, EPI_ISL_1816962, EPI_ISL_1816963, EPI_ISL_1816964, EPI_ISL_1816965, EPI_ISL_1816966, EPI_ISL_1816967, EPI_ISL_1816968, EPI_ISL_1816969, EPI_ISL_1816970, EPI_ISL_1816971, EPI_ISL_1816972, EPI_ISL_1816973, EPI_ISL_1897601, EPI_ISL_1897602, EPI_ISL_1897603, EPI_ISL_1897604, EPI_ISL_1897605, EPI_ISL_1897606, EPI_ISL_1897607, EPI_ISL_1897608, EPI_ISL_1897609, EPI_ISL_1897610, EPI_ISL_1897611, EPI_ISL_1897612, EPI_ISL_1897613, EPI_ISL_1897614, EPI_ISL_1897615, EPI_ISL_1897616, EPI_ISL_1897617, EPI_ISL_1897618, EPI_ISL_1897619, EPI_ISL_1897620, EPI_ISL_1897621, EPI_ISL_1897622, EPI_ISL_1897623, EPI_ISL_1897624, EPI_ISL_1897625, EPI_ISL_1897626, EPI_ISL_1897627, EPI_ISL_1897628, EPI_ISL_1897629, EPI_ISL_1897630, EPI_ISL_1897631, EPI_ISL_1897632, EPI_ISL_1897633, EPI_ISL_1897634, EPI_ISL_1897635, EPI_ISL_1897636, EPI_ISL_1897637, EPI_ISL_1897638, EPI_ISL_1897639, EPI_ISL_1897640, EPI_ISL_1897641, EPI_ISL_1914639, EPI_ISL_1914640, EPI_ISL_1914641, EPI_ISL_1914642, EPI_ISL_1914643, EPI_ISL_1914644, EPI_ISL_1914645, EPI_ISL_1914646, EPI_ISL_1914647, EPI_ISL_1914648, EPI_ISL_1914649, EPI_ISL_1914650, EPI_ISL_1914651, EPI_ISL_1914652, EPI_ISL_1914653, EPI_ISL_1914654, EPI_ISL_1914655, EPI_ISL_1914656, EPI_ISL_1914657, EPI_ISL_1914658, EPI_ISL_1914659, EPI_ISL_1914660, EPI_ISL_1914661, EPI_ISL_1914662			
see above	National Public Health Laboratory, National Centre for Infectious Diseases	National Public Health Laboratory, National Centre for Infectious Diseases	Tze Minn Mak, Zhenyang Zhou, Grace Jie Yin Ngan, Royce Ang, Lin Cui, Raymond Tzer Pin Lin
EPI_ISL_1969690	Singapore General Hospital	Department of Microbiology	Nurdyana Abdul Rahman, Kenneth Xin Long Chan, Suphavilai Chayaporn, Kun Lee Lim, Sui Sin Goh, Kian Sing Chan, Lynette Oon, Kern Rei Chng, James Sim Heng Chiak, Niranjan Nagarajan, Karrie Ko
EPI_ISL_2349709, EPI_ISL_2349710, EPI_ISL_2349711, EPI_ISL_2349712, EPI_ISL_2349713, EPI_ISL_2349714, EPI_ISL_2349715, EPI_ISL_2349716, EPI_ISL_2349717, EPI_ISL_2349718, EPI_ISL_2349719, EPI_ISL_2349720, EPI_ISL_2349721, EPI_ISL_2349722, EPI_ISL_2349723, EPI_ISL_2349724, EPI_ISL_2349725, EPI_ISL_2349726, EPI_ISL_2349727, EPI_ISL_2349728, EPI_ISL_2349729, EPI_ISL_2349730, EPI_ISL_2349731, EPI_ISL_2349732, EPI_ISL_2349733, EPI_ISL_2349734, EPI_ISL_2349735, EPI_ISL_2349736, EPI_ISL_2349737, EPI_ISL_2349738, EPI_ISL_2349739, EPI_ISL_2349740, EPI_ISL_2349741, EPI_ISL_2349742, EPI_ISL_2349743, EPI_ISL_2349744, EPI_ISL_2349745, EPI_ISL_2349746, EPI_ISL_2349747, EPI_ISL_2349748, EPI_ISL_2349749, EPI_ISL_2349750, EPI_ISL_2349751, EPI_ISL_2349752, EPI_ISL_2349753, EPI_ISL_2349754, EPI_ISL_2349755, EPI_ISL_2349756, EPI_ISL_2349757, EPI_ISL_2349758, EPI_ISL_2349759, EPI_ISL_2349760, EPI_ISL_2349761, EPI_ISL_2349762, EPI_ISL_2349763, EPI_ISL_2349764, EPI_ISL_2349765, EPI_ISL_2349766, EPI_ISL_2349767, EPI_ISL_2349768, EPI_ISL_2349769, EPI_ISL_2349770, EPI_ISL_2349771, EPI_ISL_2349772, EPI_ISL_2349773, EPI_ISL_2349774, EPI_ISL_2349775, EPI_ISL_2349776, EPI_ISL_2349777, EPI_ISL_2349778, EPI_ISL_2349779, EPI_ISL_2349780, EPI_ISL_2349781, EPI_ISL_2349782, EPI_ISL_2349783, EPI_ISL_2349784, EPI_ISL_2349785, EPI_ISL_2349786, EPI_ISL_2349787, EPI_ISL_2349788, EPI_ISL_2349789, EPI_ISL_2349790, EPI_ISL_2349791			
see above	National Public Health Laboratory, National Centre for Infectious Diseases	National Public Health Laboratory, National Centre for Infectious Diseases	Tze Minn Mak, Zhenyang Zhou, Grace Jie Yin Ngan, Royce Ang, Lin Cui, Raymond Tzer Pin Lin
EPI_ISL_2349792, EPI_ISL_2349793, EPI_ISL_2349794, EPI_ISL_2349795, EPI_ISL_2349796, EPI_ISL_2349797, EPI_ISL_2349798, EPI_ISL_2349799, EPI_ISL_2349800, EPI_ISL_2349801, EPI_ISL_2349802, EPI_ISL_2349803, EPI_ISL_2349804, EPI_ISL_2349805, EPI_ISL_2349806, EPI_ISL_2349807, EPI_ISL_2349808, EPI_ISL_2349809, EPI_ISL_2349810, EPI_ISL_2349811, EPI_ISL_2349812, EPI_ISL_2349813, EPI_ISL_2349814, EPI_ISL_2349815, EPI_ISL_2349816, EPI_ISL_2349817, EPI_ISL_2349818, EPI_ISL_2349819, EPI_ISL_2349820, EPI_ISL_2349821, EPI_ISL_2349822, EPI_ISL_2349823, EPI_ISL_2349824, EPI_ISL_2349825, EPI_ISL_2349826, EPI_ISL_2349827, EPI_ISL_2349828, EPI_ISL_2349829, EPI_ISL_2349830, EPI_ISL_2349831, EPI_ISL_2349832, EPI_ISL_2349833, EPI_ISL_2349834, EPI_ISL_2349835, EPI_ISL_2349836, EPI_ISL_2349837, EPI_ISL_2349838, EPI_ISL_2349839, EPI_ISL_2349840, EPI_ISL_2349841, EPI_ISL_2349842,			

EPI_ISL_2349843, EPI_ISL_2349844, EPI_ISL_2349845, EPI_ISL_2349846, EPI_ISL_2349847, EPI_ISL_2349848, EPI_ISL_2349849, EPI_ISL_2349850, EPI_ISL_2349851, EPI_ISL_2349852, EPI_ISL_2349853, EPI_ISL_2349854, EPI_ISL_2349855, EPI_ISL_2349856, EPI_ISL_2349857, EPI_ISL_2349858, EPI_ISL_2349859, EPI_ISL_2349860, EPI_ISL_2349861, EPI_ISL_2349862, EPI_ISL_2349863, EPI_ISL_2349864, EPI_ISL_2349865, EPI_ISL_2349866, EPI_ISL_2349867, EPI_ISL_2349868, EPI_ISL_2349869, EPI_ISL_2349870, EPI_ISL_2349871, EPI_ISL_2349872, EPI_ISL_2349873, EPI_ISL_2349874, EPI_ISL_2349875, EPI_ISL_2349876, EPI_ISL_2349877, EPI_ISL_2349878

see above	National Public Health Laboratory, National Centre for Infectious Diseases	National Public Health Laboratory, National Centre for Infectious Diseases	Tze Minn Mak, Zhenyang Zhou, Royce Ang, Lin Cui, Raymond Tzer Pin Lin
EPI_ISL_2464555, EPI_ISL_2464556, EPI_ISL_2464557, EPI_ISL_2464558, EPI_ISL_2464559	Singapore General Hospital	Department of Microbiology	Nurdyana Abdul Rahman, Kenneth Xin Long Chan, Chayaporn Suphavilai, Kun Lee Lim, Sui Sin Goh, Kian Sing Chan, Lynette Oon, Kern Rei Chng, James Sim Heng Chiak, Niranjan Nagarajan, Karrie Ko
EPI_ISL_2464560, EPI_ISL_2464561, EPI_ISL_2464562, EPI_ISL_2464563, EPI_ISL_2464564	Singapore General Hospital	Singapore General Hospital	Nurdyana Abdul Rahman, Kenneth Xin Long Chan, Chayaporn Suphavilai, Kun Lee Lim, Sui Sin Goh, Kian Sing Chan, Lynette Oon, Kern Rei Chng, James Sim Heng Chiak, Niranjan Nagarajan, Karrie Ko

EPI_ISL_2508617, EPI_ISL_2508618, EPI_ISL_2508619, EPI_ISL_2508620, EPI_ISL_2508621, EPI_ISL_2508622, EPI_ISL_2508623, EPI_ISL_2508624, EPI_ISL_2508625, EPI_ISL_2508626, EPI_ISL_2508627, EPI_ISL_2508628, EPI_ISL_2508629, EPI_ISL_2508630, EPI_ISL_2508631, EPI_ISL_2508632, EPI_ISL_2508633, EPI_ISL_2508634, EPI_ISL_2508635, EPI_ISL_2508636, EPI_ISL_2508637, EPI_ISL_2508638, EPI_ISL_2508639, EPI_ISL_2508640, EPI_ISL_2508641, EPI_ISL_2508642, EPI_ISL_2508643, EPI_ISL_2508644, EPI_ISL_2508645, EPI_ISL_2508646, EPI_ISL_2508647, EPI_ISL_2508648, EPI_ISL_2508649, EPI_ISL_2508650, EPI_ISL_2508651, EPI_ISL_2508652, EPI_ISL_2508653, EPI_ISL_2508654, EPI_ISL_2508655, EPI_ISL_2508656, EPI_ISL_2508657, EPI_ISL_2508658, EPI_ISL_2508659, EPI_ISL_2508660, EPI_ISL_2508661, EPI_ISL_2508662, EPI_ISL_2508663, EPI_ISL_2508664, EPI_ISL_2508665, EPI_ISL_2508666, EPI_ISL_2508667, EPI_ISL_2508668, EPI_ISL_2508669, EPI_ISL_2508670, EPI_ISL_2508671, EPI_ISL_2508672, EPI_ISL_2508673, EPI_ISL_2508674, EPI_ISL_2508675, EPI_ISL_2508676, EPI_ISL_2508677, EPI_ISL_2508678, EPI_ISL_2508679, EPI_ISL_2508680, EPI_ISL_2508681, EPI_ISL_2508682, EPI_ISL_2508683, EPI_ISL_2508684, EPI_ISL_2508685, EPI_ISL_2508686, EPI_ISL_2508687, EPI_ISL_2508688, EPI_ISL_2508689, EPI_ISL_2508690, EPI_ISL_2508691, EPI_ISL_2508692, EPI_ISL_2508693, EPI_ISL_2508694, EPI_ISL_2508695, EPI_ISL_2508696, EPI_ISL_2508697, EPI_ISL_2508698, EPI_ISL_2508699, EPI_ISL_2508700, EPI_ISL_2508701, EPI_ISL_2508702, EPI_ISL_2508703, EPI_ISL_2508704, EPI_ISL_2508705, EPI_ISL_2508706, EPI_ISL_2508707, EPI_ISL_2508708, EPI_ISL_2508709, EPI_ISL_2508710, EPI_ISL_2508711, EPI_ISL_2508712, EPI_ISL_2508713, EPI_ISL_2508714, EPI_ISL_2508715, EPI_ISL_2508716, EPI_ISL_2508717, EPI_ISL_2508718, EPI_ISL_2508719, EPI_ISL_2508720, EPI_ISL_2508721, EPI_ISL_2508722, EPI_ISL_2508723, EPI_ISL_2508724, EPI_ISL_2508725, EPI_ISL_2508726, EPI_ISL_2508727, EPI_ISL_2508728, EPI_ISL_2508729, EPI_ISL_2508730, EPI_ISL_2508731, EPI_ISL_2508732, EPI_ISL_2508733, EPI_ISL_2508734, EPI_ISL_2508735, EPI_ISL_2508736, EPI_ISL_2508737, EPI_ISL_2508738, EPI_ISL_2508739, EPI_ISL_2508740, EPI_ISL_2508741, EPI_ISL_2508742, EPI_ISL_2508743, EPI_ISL_2508744, EPI_ISL_2508745, EPI_ISL_2508746, EPI_ISL_2508747, EPI_ISL_2508748, EPI_ISL_2508749, EPI_ISL_2508750, EPI_ISL_2508751, EPI_ISL_2508752, EPI_ISL_2508753, EPI_ISL_2508754, EPI_ISL_2508755, EPI_ISL_2508756, EPI_ISL_2508757, EPI_ISL_2508758, EPI_ISL_2508759, EPI_ISL_2508760, EPI_ISL_2508761, EPI_ISL_2508762, EPI_ISL_2508763, EPI_ISL_2508764, EPI_ISL_2508765, EPI_ISL_2508766, EPI_ISL_2508767, EPI_ISL_2508768, EPI_ISL_2508769, EPI_ISL_2508770, EPI_ISL_2508771, EPI_ISL_2508772, EPI_ISL_2508773, EPI_ISL_2508774, EPI_ISL_2508775, EPI_ISL_2508776, EPI_ISL_2508777, EPI_ISL_2508778, EPI_ISL_2508779, EPI_ISL_2508780, EPI_ISL_2508781, EPI_ISL_2508782, EPI_ISL_2508783, EPI_ISL_2508784, EPI_ISL_2508785, EPI_ISL_2508786, EPI_ISL_2508787, EPI_ISL_2508788, EPI_ISL_2508789, EPI_ISL_2508790, EPI_ISL_2508791, EPI_ISL_2508792, EPI_ISL_2508793, EPI_ISL_2508794, EPI_ISL_2508795, EPI_ISL_2508796, EPI_ISL_2508797, EPI_ISL_2508798, EPI_ISL_2508799, EPI_ISL_2508800, EPI_ISL_2508801, EPI_ISL_2508802, EPI_ISL_2508803, EPI_ISL_2508804, EPI_ISL_2508805, EPI_ISL_2508806, EPI_ISL_2508807, EPI_ISL_2508808, EPI_ISL_2508809, EPI_ISL_2508810, EPI_ISL_2508811, EPI_ISL_2508812, EPI_ISL_2508813, EPI_ISL_2508814, EPI_ISL_2508815, EPI_ISL_2508816, EPI_ISL_2508817, EPI_ISL_2508818, EPI_ISL_2508819, EPI_ISL_2508820, EPI_ISL_2508821, EPI_ISL_2508822, EPI_ISL_2508823, EPI_ISL_2508824, EPI_ISL_2508825, EPI_ISL_2508826, EPI_ISL_2508827, EPI_ISL_2508828, EPI_ISL_2508829, EPI_ISL_2508830, EPI_ISL_2508831, EPI_ISL_2508832, EPI_ISL_2508833, EPI_ISL_2508834, EPI_ISL_2508835, EPI_ISL_2508836, EPI_ISL_2508837, EPI_ISL_2508838, EPI_ISL_2508839, EPI_ISL_2508840, EPI_ISL_2508841, EPI_ISL_2508842, EPI_ISL_2508843, EPI_ISL_2508844, EPI_ISL_2508845, EPI_ISL_2508846, EPI_ISL_2508847, EPI_ISL_2508848, EPI_ISL_2508849, EPI_ISL_2508850, EPI_ISL_2508851, EPI_ISL_2508852, EPI_ISL_2508853, EPI_ISL_2508854, EPI_ISL_2508855, EPI_ISL_2508856, EPI_ISL_2508857, EPI_ISL_2508858, EPI_ISL_2508859, EPI_ISL_2508860, EPI_ISL_2508861, EPI_ISL_2508862, EPI_ISL_2508863, EPI_ISL_2508864, EPI_ISL_2508865, EPI_ISL_2508866, EPI_ISL_2508867, EPI_ISL_2508868, EPI_ISL_2508869, EPI_ISL_2508870, EPI_ISL_2508871, EPI_ISL_2508872, EPI_ISL_2508873, EPI_ISL_2508874, EPI_ISL_2508875, EPI_ISL_2508876, EPI_ISL_2508877, EPI_ISL_2508878, EPI_ISL_2508879, EPI_ISL_2508880, EPI_ISL_2508881, EPI_ISL_2508882, EPI_ISL_2508883, EPI_ISL_2508884, EPI_ISL_2508885, EPI_ISL_2508886, EPI_ISL_2508887, EPI_ISL_2508888, EPI_ISL_2508889, EPI_ISL_2508890, EPI_ISL_2508891, EPI_ISL_2508892, EPI_ISL_2508893, EPI_ISL_2508894, EPI_ISL_2508895, EPI_ISL_2508896, EPI_ISL_2508897, EPI_ISL_2508898, EPI_ISL_2508899, EPI_ISL_2508900, EPI_ISL_2508901, EPI_ISL_2508902, EPI_ISL_2508903, EPI_ISL_2508904, EPI_ISL_2508905, EPI_ISL_2508906, EPI_ISL_2508907, EPI_ISL_2508908, EPI_ISL_2508909, EPI_ISL_2508910, EPI_ISL_2508911, EPI_ISL_2508912, EPI_ISL_2508913, EPI_ISL_2508914, EPI_ISL_2508915, EPI_ISL_2508916, EPI_ISL_2508917, EPI_ISL_2508918, EPI_ISL_2508919, EPI_ISL_2508920, EPI_ISL_2508921, EPI_ISL_2508922, EPI_ISL_2508923, EPI_ISL_2508924, EPI_ISL_2508925, EPI_ISL_2508926, EPI_ISL_2508927, EPI_ISL_2508928, EPI_ISL_2508929, EPI_ISL_2508930, EPI_ISL_2508931, EPI_ISL_2508932, EPI_ISL_2508933, EPI_ISL_2508934, EPI_ISL_2508935, EPI_ISL_2508936, EPI_ISL_2508937, EPI_ISL_2508938, EPI_ISL_2508939, EPI_ISL_2508940, EPI_ISL_2508941, EPI_ISL_2508942, EPI_ISL_2508943, EPI_ISL_2508944, EPI_ISL_2508945, EPI_ISL_2508946, EPI_ISL_2508947, EPI_ISL_2508948, EPI_ISL_2508949, EPI_ISL_2508950, EPI_ISL_2508951, EPI_ISL_2508952, EPI_ISL_2508953, EPI_ISL_2508954, EPI_ISL_2508955, EPI_ISL_2508956, EPI_ISL_2508957, EPI_ISL_2508958, EPI_ISL_2508959, EPI_ISL_2508960, EPI_ISL_2508961, EPI_ISL_2508962, EPI_ISL_2508963, EPI_ISL_2508964, EPI_ISL_2508965, EPI_ISL_2508966, EPI_ISL_2508967, EPI_ISL_2508968, EPI_ISL_2508969, EPI_ISL_2508970, EPI_ISL_2508971, EPI_ISL_2508972, EPI_ISL_2508973, EPI_ISL_2508974, EPI_ISL_2508975, EPI_ISL_2508976, EPI_ISL_2508977, EPI_ISL_2508978, EPI_ISL_2508979, EPI_ISL_2508980, EPI_ISL_2508981, EPI_ISL_2508982, EPI_ISL_2508983, EPI_ISL_2508984, EPI_ISL_2508985, EPI_ISL_2508986, EPI_ISL_2508987, EPI_ISL_2508988, EPI_ISL_2508989, EPI_ISL_2508990, EPI_ISL_2508991, EPI_ISL_2508992, EPI_ISL_2508993, EPI_ISL_2508994, EPI_ISL_2508995, EPI_ISL_2508996, EPI_ISL_2508997, EPI_ISL_2508998, EPI_ISL_2508999, EPI_ISL_2509000, EPI_ISL_2509001, EPI_ISL_2509002, EPI_ISL_2509003, EPI_ISL_2509004, EPI_ISL_2509005, EPI_ISL_2509006, EPI_ISL_2509007, EPI_ISL_2509008, EPI_ISL_2509009, EPI_ISL_2509010, EPI_ISL_2509011, EPI_ISL_2509012, EPI_ISL_2509013, EPI_ISL_2509014, EPI_ISL_2509015, EPI_ISL_2509016, EPI_ISL_2509017, EPI_ISL_2509018, EPI_ISL_2509019, EPI_ISL_2509020, EPI_ISL_2509021, EPI_ISL_2509022, EPI_ISL_2509023, EPI_ISL_2509024, EPI_ISL_2509025, EPI_ISL_2509026, EPI_ISL_2509027, EPI_ISL_2509028, EPI_ISL_2509029, EPI_ISL_2509030, EPI_ISL_2509031, EPI_ISL_2509032, EPI_ISL_2509033, EPI_ISL_2509034, EPI_ISL_2509035, EPI_ISL_2509036, EPI_ISL_2509037, EPI_ISL_2509038, EPI_ISL_2509039, EPI_ISL_2509040, EPI_ISL_2509041, EPI_ISL_2509042, EPI_ISL_2509043, EPI_ISL_2509044, EPI_ISL_2509045, EPI_ISL_2509046, EPI_ISL_2509047, EPI_ISL_2509048, EPI_ISL_2509049, EPI_ISL_2509050, EPI_ISL_2509051, EPI_ISL_2509052, EPI_ISL_2509053, EPI_ISL_2509054, EPI_ISL_2509055, EPI_ISL_2509056, EPI_ISL_2509057, EPI_ISL_2509058, EPI_ISL_2509059, EPI_ISL_2509060, EPI_ISL_2509061, EPI_ISL_2509062, EPI_ISL_2509063, EPI_ISL_2509064, EPI_ISL_2509065, EPI_ISL_2509066, EPI_ISL_2509067, EPI_ISL_2509068, EPI_ISL_2509069, EPI_ISL_2509070, EPI_ISL_2509071, EPI_ISL_2509072, EPI_ISL_2509073, EPI_ISL_2509074, EPI_ISL_2509075, EPI_ISL_2509076, EPI_ISL_2509077, EPI_ISL_2509078, EPI_ISL_2509079, EPI_ISL_2509080, EPI_ISL_2509081, EPI_ISL_2509082, EPI_ISL_2509083, EPI_ISL_2509084, EPI_ISL_2509085, EPI_ISL_2509086, EPI_ISL_2509087, EPI_ISL_2509088, EPI_ISL_2509089, EPI_ISL_2509090, EPI_ISL_2509091, EPI_ISL_2509092, EPI_ISL_2509093, EPI_ISL_2509094, EPI_ISL_2509095, EPI_ISL_2509096, EPI_ISL_2509097, EPI_ISL_2509098, EPI_ISL_2509099, EPI_ISL_2509100, EPI_ISL_2509101, EPI_ISL_2509102, EPI_ISL_2509103, EPI_ISL_2509104, EPI_ISL_2509105, EPI_ISL_2509106, EPI_ISL_2509107, EPI_ISL_2509108, EPI_ISL_2509109, EPI_ISL_2509110, EPI_ISL_2509111

see above	National Public Health Laboratory, National Centre for Infectious Diseases	National Public Health Laboratory, National Centre for Infectious Diseases	Tze Minn Mak, Zhenyang Zhou, Royce Ang, Lin Cui, Raymond Tzer Pin Lin
EPI_ISL_2566444, EPI_ISL_2566445	Singapore General Hospital	Department of Microbiology	Nurdyana Abdul Rahman, Kenneth Xin Long Chan, Chayaporn Suphavilai, Kun Lee Lim, Sui Sin Goh, Kian Sing Chan, Lynette Oon, Kern Rei Chng, James Sim Heng Chiak, Niranjan Nagarajan, Karrie Ko

EPI_ISL_2621885, EPI_ISL_2621886, EPI_ISL_2621887, EPI_ISL_2621888, EPI_ISL_2621889, EPI_ISL_2621890, EPI_ISL_2621891, EPI_ISL_2621892, EPI_ISL_2621893, EPI_ISL_2621894, EPI_ISL_2621895, EPI_ISL_2621896, EPI_ISL_2621897, EPI_ISL_2621898, EPI_ISL_2621899, EPI_ISL_2621900, EPI_ISL_2621901, EPI_ISL_2621902, EPI_ISL_2621903, EPI_ISL_2621904, EPI_ISL_2621905, EPI_ISL_2621906, EPI_ISL_2621907, EPI_ISL_2621908, EPI_ISL_2621909, EPI_ISL_2621910, EPI_ISL_2621911, EPI_ISL_2621912, EPI_ISL_2621913, EPI_ISL_2621914, EPI_ISL_2621915, EPI_ISL_2621916, EPI_ISL_2621917, EPI_ISL_2621918, EPI_ISL_2621919, EPI_ISL_2621920, EPI_ISL_2621921, EPI_ISL_2621922, EPI_ISL_2621923, EPI_ISL_2621924, EPI_ISL_2621925, EPI_ISL_2621926, EPI_ISL_2621927, EPI_ISL_2621928, EPI_ISL_2621929, EPI_ISL_2621930, EPI_ISL_2621931, EPI_ISL_2621932, EPI_ISL_2621933, EPI_ISL_2621934, EPI_ISL_2621935, EPI_ISL_2621936, EPI_ISL_2621937, EPI_ISL_2621938, EPI_ISL_2621939, EPI_ISL_2621940, EPI_ISL_2621941, EPI_ISL_2621942, EPI_ISL_2621943, EPI_ISL_2621944, EPI_ISL_2621945, EPI_ISL_2621946, EPI_ISL_2621947, EPI_ISL_2621948, EPI_ISL_2621949, EPI_ISL_2621950, EPI_ISL_2621951, EPI_ISL_2621952, EPI_ISL_2621953, EPI_ISL_2621954, EPI_ISL_2621955, EPI_ISL_2621956, EPI_ISL_2621957, EPI_ISL_2621958, EPI_ISL_2621959, EPI_ISL_2621960, EPI_ISL_2621961, EPI_ISL_2621962, EPI_ISL_2621963, EPI_ISL_2621964

see above	National Public Health Laboratory, National Centre for Infectious Diseases	National Public Health Laboratory, National Centre for Infectious Diseases	Tze Minn Mak, Zhenyang Zhou, Royce Ang, Lin Cui, Raymond Tzer Pin Lin

EPI_ISL_2627533, EPI_ISL_2627534, EPI_ISL_2627535, EPI_ISL_2627536, EPI_ISL_2627537, EPI_ISL_2627538, EPI_ISL_2627539, EPI_ISL_2627540, EPI_ISL_2627541, EPI_ISL_2627542, EPI_ISL_2627543, EPI_ISL_2627544, EPI_ISL_2627545, EPI_ISL_2627546, EPI_ISL_2627547, EPI_ISL_2627548, EPI_ISL_2627549, EPI_ISL_2627550, EPI_ISL_2627551, EPI_ISL_2627552, EPI_ISL_2627553, EPI_ISL_2627554, EPI_ISL_2627555, EPI_ISL_2627556, EPI_ISL_2627557, EPI_ISL_2627558, EPI_ISL_2627559, EPI_ISL_2627560, EPI_ISL_2627561, EPI_ISL_2627562, EPI_ISL_2627563, EPI_ISL_2627564, EPI_ISL_2627565, EPI_ISL_2627566, EPI_ISL_2627567, EPI_ISL_2627568, EPI_ISL_2627569, EPI_ISL_2627570, EPI_ISL_2627571, EPI_ISL_2627572, EPI_ISL_2627573, EPI_ISL_2627574, EPI_ISL_2627575, EPI_ISL_2627576, EPI_ISL_2627577, EPI_ISL_2627578, EPI_ISL_2627579, EPI_ISL_2627580, EPI_ISL_2710081, EPI_ISL_2710082, EPI_ISL_2710083, EPI_ISL_2710084, EPI_ISL_2710085, EPI_ISL_2710086, EPI_ISL_2710087, EPI_ISL_2710088, EPI_ISL_2710089, EPI_ISL_2710090, EPI_ISL_2710091, EPI_ISL_2710092, EPI_ISL_2710093, EPI_ISL_2710094, EPI_ISL_2710095, EPI_ISL_2710096, EPI_ISL_2710097, EPI_ISL_2710098, EPI_ISL_2710099, EPI_ISL_2710100, EPI_ISL_2710101, EPI_ISL_2710102, EPI_ISL_2710103, EPI_ISL_2710104, EPI_ISL_2710105, EPI_ISL_2710106, EPI_ISL_2710107, EPI_ISL_2710108, EPI_ISL_2710109, EPI_ISL_2710110, EPI_ISL_2710111, EPI_ISL_2710112, EPI_ISL_2710113, EPI_ISL_2710114, EPI_ISL_2710115, EPI_ISL_2710116, EPI_ISL_2710117, EPI_ISL_2710118, EPI_ISL_2710119, EPI_ISL_2710120, EPI_ISL_2710121, EPI_ISL_2710122, EPI_ISL_2710123, EPI_ISL_2710124, EPI_ISL_2710125, EPI_ISL_2710126, EPI_ISL_2710127, EPI_ISL_2710128, EPI_ISL_2710129, EPI_ISL_2710130, EPI_ISL_2710131, EPI_ISL_2710132, EPI_ISL_2710133, EPI_ISL_2710134, EPI_ISL_2710135, EPI_ISL_2710136, EPI_ISL_2710137, EPI_ISL_2710138, EPI_ISL_2710139, EPI_ISL_2710140, EPI_ISL_2710141, EPI_ISL_2710142, EPI_ISL_2710143, EPI_ISL_2710144, EPI_ISL_2710145, EPI_ISL_2710146, EPI_ISL_2710147, EPI_ISL_2710148, EPI_ISL_2710149, EPI_ISL_2710150, EPI_ISL_2710151, EPI_ISL_2710152, EPI_ISL_2710153, EPI_ISL_2710154, EPI_ISL_2710155, EPI_ISL_2710156, EPI_ISL_2710157, EPI_ISL_2710158, EPI_ISL_2710159, EPI_ISL_2710160, EPI_ISL_2710161, EPI_ISL_2710162, EPI_ISL_2710163, EPI_ISL_2710164, EPI_ISL_2710165, EPI_ISL_2710166, EPI_ISL_2710167, EPI_ISL_2710168, EPI_ISL_2710169, EPI_ISL_2710170, EPI_ISL_2710171

see above	National Public Health Laboratory, National Centre for Infectious Diseases	National Public Health Laboratory, National Centre for Infectious Diseases	Tze Minn Mak, Katherine Ching, Zhenyang Zhou, Royce Ang, Lin Cui, Raymond Tzer Pin Lin

EPI_ISL_2790295, EPI_ISL_2790296, EPI_ISL_2790297, EPI_ISL_2790298, EPI_ISL_2790299, EPI_ISL_2790300, EPI_ISL_2790301, EPI_ISL_2790302, EPI_ISL_2790303, EPI_ISL_2790304, EPI_ISL_2790305

see above	National Public Health Laboratory, National Centre for Infectious Diseases	National Public Health Laboratory, National Centre for Infectious Diseases	Tze Minn Mak, Zhenyang Zhou, Royce Ang, Lin Cui, Raymond Tzer Pin Lin

EPI_ISL_2790332, EPI_ISL_2790333, EPI_ISL_2790334, EPI_ISL_2790335, EPI_ISL_2790336, EPI_ISL_2790337, EPI_ISL_2790338, EPI_ISL_2790339, EPI_ISL_2790340, EPI_ISL_2790341, EPI_ISL_2790342, EPI_ISL_2790343, EPI_ISL_2790344, EPI_ISL_2790345, EPI_ISL_2790346, EPI_ISL_2790347, EPI_ISL_2790348, EPI_ISL_2790349, EPI_ISL_2790350, EPI_ISL_2790351, EPI_ISL_2790352, EPI_ISL_2790353, EPI_ISL_2790354, EPI_ISL_2790355, EPI_ISL_2790356, EPI_ISL_2790357, EPI_ISL_2790358, EPI_ISL_2790359, EPI_ISL_2790360, EPI_ISL_2790361, EPI_ISL_2790362, EPI_ISL_2790363, EPI_ISL_2790364, EPI_ISL_2790365, EPI_ISL_2790366, EPI_ISL_2790367, EPI_ISL_2790368, EPI_ISL_2790369, EPI_ISL_2790370, EPI_ISL_2790371, EPI_ISL_2790372, EPI_ISL_2790373, EPI_ISL_2790374, EPI_ISL_2790375, EPI_ISL_2790376, EPI_ISL_2790377, EPI_ISL_2790378, EPI_ISL_2790379, EPI_ISL_2790380, EPI_ISL_2790381, EPI_ISL_2790382, EPI_ISL_2790383, EPI_ISL_2790384, EPI_ISL_2790385, EPI_ISL_2790386, EPI_ISL_2790387

see above	National Public Health Laboratory, National Centre for Infectious Diseases	National Public Health Laboratory, National Centre for Infectious Diseases	Tze Minn Mak, Katherine Ching, Zhenyang Zhou, Royce Ang, Lin Cui, Raymond Tzer Pin Lin

EPI_ISL_2812146, EPI_ISL_2812147, EPI_ISL_2812148, EPI_ISL_2812149, EPI_ISL_2812150, EPI_ISL_2812151, EPI_ISL_2812152, EPI_ISL_2812153, EPI_ISL_2812154, EPI_ISL_2812155, EPI_ISL_2812156

see above	Singapore General Hospital	Department of Microbiology	Nurdyana Abdul Rahman, Kenneth Xin Long Chan, Chayaporn Suphavilai, Kun Lee Lim, Sui Sin Goh, Kian Sing Chan, Lynette Oon, Kern Rei Chng, James Sim Heng Chiak, Niranjan Nagarajan, Karrie Ko

EPI_ISL_2820263, EPI_ISL_2820264, EPI_ISL_2820265, EPI_ISL_2820266, EPI_ISL_2820267, EPI_ISL_2820268, EPI_ISL_2820269, EPI_ISL_2820270, EPI_ISL_2820271, EPI_ISL_2820272, EPI_ISL_2820273, EPI_ISL_2820274, EPI_ISL_2820275, EPI_ISL_2820276, EPI_ISL_2820277, EPI_ISL_2820278, EPI_ISL_2820279, EPI_ISL_2820280, EPI_ISL_2820281, EPI_ISL_2820282, EPI_ISL_2820283, EPI_ISL_2820284, EPI_ISL_2820285, EPI_ISL_2820286, EPI_ISL_2820287, EPI_ISL_2887795

see above	National Public Health Laboratory, National Centre for	National Public Health Laboratory, National Centre for	Tze Minn Mak, Katherine Ching, Zhenyang Zhou, Royce Ang, Lin Cui, Raymond Tzer Pin Lin

	Infectious Diseases	Infectious Diseases	
EPI_ISL_406973	Singapore General Hospital	National Public Health Laboratory	Mak, TM; Octavia S; Chavatte JM; Zhou, ZY; Cui, L; Lin, RTP
EPI_ISL_407987	Singapore General Hospital	Programme in Emerging Infectious Diseases, Duke-NUS Medical School	Danielle E Anderson, Martin Linster, Yan Zhuang, Jayanthi Jayakumar, Kian Sing Chan, Lynette LE Oon, Jenny GH Low, Yvonne CF Su, Linfa Wang, Gavin JD Smith
EPI_ISL_407988	National Centre for Infectious Diseases	Programme in Emerging Infectious Diseases, Duke-NUS Medical School	Danielle E Anderson, Martin Linster, Yan Zhuang, Jayanthi Jayakumar, David CB Lye, Yee Sin Leo, Barnaby E Young, Yvonne CF Su, Linfa Wang, Gavin JD Smith
EPI_ISL_410535	National Centre for Infectious Diseases	Programme in Emerging Infectious Diseases, Duke-NUS Medical School	Danielle E Anderson, Martin Linster, Yan Zhuang, Jayanthi Jayakumar, David CB Lye, Yee Sin Leo, Barnaby E Young, Yvonne CF Su, Gavin JD Smith
EPI_ISL_410536, EPI_ISL_410537	Singapore General Hospital, Molecular Laboratory, Division of Pathology	Programme in Emerging Infectious Diseases, Duke-NUS Medical School	Danielle E Anderson, Martin Linster, Yan Zhuang, Jayanthi Jayakumar, Kian Sing Chan, Lynette LE Oon, Shirin Kalimuddin, Jenny GH Low, Yvonne CF Su, Gavin JD Smith
EPI_ISL_410713, EPI_ISL_410714, EPI_ISL_410715	National Public Health Laboratory, National Centre for Infectious Diseases	National Public Health Laboratory, National Centre for Infectious Diseases	Octavia S, Mak TM, Cui L, Lin RTP
EPI_ISL_410716	National Public Health Laboratory, National Centre for Infectious Diseases	National Centre for Infectious Diseases, National Centre for Infectious Diseases	Octavia S, Mak TM, Cui L, Lin RTP
EPI_ISL_410719	National Public Health Laboratory	National Public Health Laboratory	Octavia S, Mak TM, Cui L, Lin RTP
EPI_ISL_414378	National Centre for Infectious Diseases	Programme in Emerging Infectious Diseases, Duke-NUS Medical School	Danielle E Anderson, Martin Linster, Yan Zhuang, Jayanthi Jayakumar, Louisa Sun, David CB Lye, Yee Sin Leo, Barnaby E Young, Yvonne CF Su, Gavin JD Smith
EPI_ISL_414379, EPI_ISL_414380	National Centre for Infectious Diseases	Programme in Emerging Infectious Diseases, Duke-NUS Medical School	Danielle E Anderson, Martin Linster, Yan Zhuang, Jayanthi Jayakumar, David CB Lye, Yee Sin Leo, Barnaby E Young, Yvonne CF Su, Gavin JD Smith
EPI_ISL_418992, EPI_ISL_418993, EPI_ISL_418994, EPI_ISL_418995, EPI_ISL_418996, EPI_ISL_418997, EPI_ISL_418998, EPI_ISL_418999, EPI_ISL_419000, EPI_ISL_419001	National Public Health Laboratory, National Centre for Infectious Diseases	National Public Health Laboratory, National Centre for Infectious Diseases	Mak TM, Octavia S, Cui L, Lin RTP
EPI_ISL_420099, EPI_ISL_420100, EPI_ISL_420101, EPI_ISL_420102, EPI_ISL_420103, EPI_ISL_420104, EPI_ISL_420105, EPI_ISL_420106, EPI_ISL_420107, EPI_ISL_420108, EPI_ISL_420109, EPI_ISL_420110, EPI_ISL_420111			
see above	National Centre for Infectious Diseases	Programme in Emerging Infectious Diseases, Duke-NUS Medical School	Danielle E Anderson, Martin Linster, Yan Zhuang, Jayanthi Jayakumar, David CB Lye, Yee Sin Leo, Barnaby E Young, Yvonne CF Su, Gavin JD Smith
EPI_ISL_422428, EPI_ISL_422429, EPI_ISL_422430, EPI_ISL_422431, EPI_ISL_422432, EPI_ISL_422433, EPI_ISL_422434, EPI_ISL_422435	National Public Health Laboratory, National Centre for Infectious Diseases	National Public Health Laboratory, National Centre for Infectious Diseases	Mak TM, Octavia S, Cui L, Lin RTP
EPI_ISL_428822, EPI_ISL_428823, EPI_ISL_428824, EPI_ISL_428825, EPI_ISL_428826, EPI_ISL_428827, EPI_ISL_428828, EPI_ISL_428829, EPI_ISL_428830, EPI_ISL_428831, EPI_ISL_428832, EPI_ISL_428833, EPI_ISL_428834, EPI_ISL_428835, EPI_ISL_428836, EPI_ISL_428837, EPI_ISL_428838, EPI_ISL_428839, EPI_ISL_428840, EPI_ISL_428841, EPI_ISL_428842, EPI_ISL_428843, EPI_ISL_428844, EPI_ISL_428845, EPI_ISL_428846, EPI_ISL_428847, EPI_ISL_428848, EPI_ISL_428849, EPI_ISL_428850			
see above	National Public Health Laboratory, National Centre for Infectious Diseases	National Public Health Laboratory, National Centre for Infectious Diseases	Mak TM, Octavia S, Chavatte JM, Cui L, Lin RTP
EPI_ISL_435678, EPI_ISL_435679, EPI_ISL_435680, EPI_ISL_435681, EPI_ISL_435682, EPI_ISL_435683, EPI_ISL_435684, EPI_ISL_435685, EPI_ISL_435686, EPI_ISL_435687, EPI_ISL_435688, EPI_ISL_435689, EPI_ISL_435690, EPI_ISL_435691, EPI_ISL_435692, EPI_ISL_435693, EPI_ISL_435694, EPI_ISL_435695, EPI_ISL_435696, EPI_ISL_435697, EPI_ISL_435698, EPI_ISL_435699, EPI_ISL_435700, EPI_ISL_443188, EPI_ISL_443189, EPI_ISL_443190, EPI_ISL_443191, EPI_ISL_443192, EPI_ISL_443193, EPI_ISL_443194, EPI_ISL_443195, EPI_ISL_443196, EPI_ISL_443197, EPI_ISL_443198, EPI_ISL_443199, EPI_ISL_443200, EPI_ISL_443201, EPI_ISL_443202, EPI_ISL_443203, EPI_ISL_443204, EPI_ISL_443205, EPI_ISL_443206, EPI_ISL_443207, EPI_ISL_443208, EPI_ISL_443209, EPI_ISL_443210, EPI_ISL_443211, EPI_ISL_443212, EPI_ISL_443213, EPI_ISL_443214, EPI_ISL_443215, EPI_ISL_443216, EPI_ISL_443217, EPI_ISL_443218, EPI_ISL_443219, EPI_ISL_443220, EPI_ISL_443221, EPI_ISL_443222, EPI_ISL_443223, EPI_ISL_443224, EPI_ISL_443225, EPI_ISL_443226, EPI_ISL_443227, EPI_ISL_443228, EPI_ISL_443229, EPI_ISL_443230, EPI_ISL_443231, EPI_ISL_443232, EPI_ISL_443233, EPI_ISL_443234, EPI_ISL_443235, EPI_ISL_443236, EPI_ISL_443237, EPI_ISL_443238, EPI_ISL_443239, EPI_ISL_443240, EPI_ISL_443241, EPI_ISL_443242, EPI_ISL_443243, EPI_ISL_443244, EPI_ISL_443245, EPI_ISL_443246, EPI_ISL_443247, EPI_ISL_443248, EPI_ISL_443249			
see above	National Public Health Laboratory, National Centre for Infectious Diseases	National Public Health Laboratory, National Centre for Infectious Diseases	Mak Tze Minn, Octavia Sophie, Chavatte Jean-Marc, Cui Lin, Lin Raymond Tzer Pin
EPI_ISL_462085, EPI_ISL_462086, EPI_ISL_462087, EPI_ISL_462088, EPI_ISL_462089	Singapore General Hospital	Department of Microbiology	Nurdyana Abdul Rahman, Kun Lee Lim, Chenhao Li, Kian Sing Chan, Lynette Oon, Kern Rei Chng, Niranjan Nagarajan, Karrie Ko
EPI_ISL_462276, EPI_ISL_462277, EPI_ISL_462278, EPI_ISL_462279, EPI_ISL_462280, EPI_ISL_462281, EPI_ISL_462282, EPI_ISL_462283, EPI_ISL_462284, EPI_ISL_462285, EPI_ISL_462286, EPI_ISL_462287, EPI_ISL_462288, EPI_ISL_462289, EPI_ISL_462290, EPI_ISL_462291, EPI_ISL_462292, EPI_ISL_462293, EPI_ISL_462294, EPI_ISL_462295, EPI_ISL_462296, EPI_ISL_462297, EPI_ISL_462298, EPI_ISL_462299, EPI_ISL_462300, EPI_ISL_462301, EPI_ISL_462302, EPI_ISL_462303, EPI_ISL_462304, EPI_ISL_462305, EPI_ISL_462306, EPI_ISL_462307, EPI_ISL_462308, EPI_ISL_462309, EPI_ISL_462310, EPI_ISL_462311, EPI_ISL_462312, EPI_ISL_462313, EPI_ISL_462314, EPI_ISL_462315, EPI_ISL_462316, EPI_ISL_462317, EPI_ISL_462318, EPI_ISL_462319, EPI_ISL_462320, EPI_ISL_462321, EPI_ISL_462322, EPI_ISL_462323, EPI_ISL_462324, EPI_ISL_462325, EPI_ISL_462326, EPI_ISL_462327, EPI_ISL_462328, EPI_ISL_462329, EPI_ISL_462330, EPI_ISL_462331, EPI_ISL_462332, EPI_ISL_462333, EPI_ISL_462334, EPI_ISL_462335, EPI_ISL_462336, EPI_ISL_462337, EPI_ISL_462338, EPI_ISL_462339, EPI_ISL_462340, EPI_ISL_462341, EPI_ISL_462342, EPI_ISL_462343, EPI_ISL_462344, EPI_ISL_462345, EPI_ISL_462346, EPI_ISL_462347, EPI_ISL_462348, EPI_ISL_462349, EPI_ISL_462350, EPI_ISL_462351, EPI_ISL_462352, EPI_ISL_462353, EPI_ISL_462354, EPI_ISL_462355, EPI_ISL_462356, EPI_ISL_462357, EPI_ISL_462358, EPI_ISL_462359, EPI_ISL_462360, EPI_ISL_462361, EPI_ISL_462362, EPI_ISL_462363, EPI_ISL_462364, EPI_ISL_462365, EPI_ISL_462366, EPI_ISL_462367, EPI_ISL_462368, EPI_ISL_462369, EPI_ISL_462370, EPI_ISL_462371, EPI_ISL_462372, EPI_ISL_462373, EPI_ISL_462374, EPI_ISL_462375, EPI_ISL_462376, EPI_ISL_462377, EPI_ISL_462378, EPI_ISL_462379, EPI_ISL_462380, EPI_ISL_462381, EPI_ISL_462382, EPI_ISL_462383, EPI_ISL_462384, EPI_ISL_462385, EPI_ISL_462386, EPI_ISL_462387, EPI_ISL_462388, EPI_ISL_462389, EPI_ISL_462390, EPI_ISL_462391, EPI_ISL_462392, EPI_ISL_462393, EPI_ISL_462394, EPI_ISL_462395, EPI_ISL_462396, EPI_ISL_462397, EPI_ISL_462398, EPI_ISL_462399, EPI_ISL_462400, EPI_ISL_462401, EPI_ISL_462402, EPI_ISL_462403, EPI_ISL_462404, EPI_ISL_462405, EPI_ISL_462406, EPI_ISL_462407, EPI_ISL_462408, EPI_ISL_462409, EPI_ISL_462410, EPI_ISL_462411, EPI_ISL_462412, EPI_ISL_462413, EPI_ISL_462414, EPI_ISL_462415, EPI_ISL_462416, EPI_ISL_462417, EPI_ISL_462418, EPI_ISL_462419, EPI_ISL_462420, EPI_ISL_462421, EPI_ISL_462422, EPI_ISL_462423, EPI_ISL_462424, EPI_ISL_462425, EPI_ISL_462426, EPI_ISL_462427, EPI_ISL_462428, EPI_ISL_462429, EPI_ISL_462430, EPI_ISL_462431, EPI_ISL_462432, EPI_ISL_462433, EPI_ISL_469080, EPI_ISL_469081, EPI_ISL_469082, EPI_ISL_469083, EPI_ISL_469084, EPI_ISL_469085, EPI_ISL_469086, EPI_ISL_469087, EPI_ISL_469088, EPI_ISL_469089, EPI_ISL_469090, EPI_ISL_469091, EPI_ISL_469092, EPI_ISL_469093, EPI_ISL_469094, EPI_ISL_469095, EPI_ISL_469096, EPI_ISL_469097, EPI_ISL_469098, EPI_ISL_469099, EPI_ISL_469100, EPI_ISL_469101, EPI_ISL_469102, EPI_ISL_469103, EPI_ISL_469104, EPI_ISL_469105, EPI_ISL_469106, EPI_ISL_469107, EPI_ISL_469108, EPI_ISL_469109, EPI_ISL_469110, EPI_ISL_469111, EPI_ISL_469112, EPI_ISL_469113, EPI_ISL_469114, EPI_ISL_469115, EPI_ISL_469116, EPI_ISL_469117, EPI_ISL_469118, EPI_ISL_469119, EPI_ISL_469120, EPI_ISL_469121, EPI_ISL_469122, EPI_ISL_469123, EPI_ISL_469124, EPI_ISL_469125, EPI_ISL_469126, EPI_ISL_469127, EPI_ISL_469128, EPI_ISL_469129, EPI_ISL_469130, EPI_ISL_469131, EPI_ISL_469132, EPI_ISL_469133, EPI_ISL_469134, EPI_ISL_469135, EPI_ISL_469136, EPI_ISL_469137, EPI_ISL_469138, EPI_ISL_469139, EPI_ISL_469140, EPI_ISL_469141, EPI_ISL_469142, EPI_ISL_469143, EPI_ISL_469144, EPI_ISL_469145, EPI_ISL_469146, EPI_ISL_469147, EPI_ISL_469148, EPI_ISL_469149, EPI_ISL_469150, EPI_ISL_469151, EPI_ISL_469152, EPI_ISL_469153, EPI_ISL_469154, EPI_ISL_469155, EPI_ISL_469274, EPI_ISL_475937, EPI_ISL_475938, EPI_ISL_475939, EPI_ISL_475940, EPI_ISL_475941, EPI_ISL_475942, EPI_ISL_475943, EPI_ISL_475944, EPI_ISL_475945, EPI_ISL_475946, EPI_ISL_475947, EPI_ISL_475948, EPI_ISL_475949, EPI_ISL_475950, EPI_ISL_475951, EPI_ISL_475952, EPI_ISL_475953, EPI_ISL_475954, EPI_ISL_475955, EPI_ISL_475956, EPI_ISL_475957, EPI_ISL_475958, EPI_ISL_475959, EPI_ISL_475960, EPI_ISL_475961, EPI_ISL_475962, EPI_ISL_475963, EPI_ISL_475964, EPI_ISL_475965, EPI_ISL_475966, EPI_ISL_475967, EPI_ISL_475968, EPI_ISL_475969, EPI_ISL_475970, EPI_ISL_475971, EPI_ISL_475972, EPI_ISL_475973, EPI_ISL_475974, EPI_ISL_475975, EPI_ISL_475976, EPI_ISL_475977, EPI_ISL_475978, EPI_ISL_475979, EPI_ISL_475980, EPI_ISL_475981, EPI_ISL_475982, EPI_ISL_475983, EPI_ISL_475984, EPI_ISL_475985, EPI_ISL_475986, EPI_ISL_475987, EPI_ISL_475988, EPI_ISL_475989, EPI_ISL_475990, EPI_ISL_475991, EPI_ISL_475992, EPI_ISL_475993, EPI_ISL_475994, EPI_ISL_475995, EPI_ISL_475996, EPI_ISL_475997, EPI_ISL_475998, EPI_ISL_475999			
see above	National Public Health Laboratory, National Centre for Infectious Diseases	National Public Health Laboratory, National Centre for Infectious Diseases	Mak TM, Octavia S, Chavatte JM, Cui L, Lin RTP
EPI_ISL_476795, EPI_ISL_476796, EPI_ISL_476797	Department of Laboratory Medicine, Tan Tock Seng Hospital	Department of Laboratory Medicine, Tan Tock Seng Hospital	Chen YYC, Zair X, Li C, Tang WY, Maurer-Stroh S, Barkham TMS, Nagarajan N, Sessions OM
EPI_ISL_476805, EPI_ISL_476806, EPI_ISL_476807, EPI_ISL_476808, EPI_ISL_476809, EPI_ISL_476810, EPI_ISL_476811, EPI_ISL_476812	Department of Laboratory Medicine Tan Tock Seng Hospital	Department of Laboratory Medicine Tan Tock Seng Hospital	Chen YYC, Zair X, Li C, Tang WY, Maurer-Stroh S, Barkham TMS, Nagarajan N, Sessions OM
EPI_ISL_476813, EPI_ISL_476814	Department of Laboratory Medicine, Tan Tock Seng Hospital	Department of Laboratory Medicine, Tan Tock Seng Hospital	Chen YYC, Zair X, Li C, Tang WY, Maurer-Stroh S, Barkham TMS, Nagarajan N, Sessions OM
EPI_ISL_476815	Department of Laboratory Medicine Tan Tock Seng Hospital	Department of Laboratory Medicine Tan Tock Seng Hospital	Chen YYC, Zair X, Li C, Tang WY, Maurer-Stroh S, Barkham TMS, Nagarajan N, Sessions OM
EPI_ISL_476816, EPI_ISL_476817, EPI_ISL_476818, EPI_ISL_476819,	Department of Laboratory Medicine, Tan Tock Seng Hospital	Department of Laboratory Medicine, Tan Tock Seng Hospital	Chen YYC, Zair X, Li C, Tang WY, Maurer-Stroh S, Barkham TMS, Nagarajan N, Sessions OM

EPI_ISL_476820, EPI_ISL_476821			
EPI_ISL_477170	Department of Laboratory, Medicine Tan Tock Seng Hospital	Department of Laboratory Medicine Tan Tock Seng Hospital	Chen YYC, Zair X, Li C, Tang WY, Maurer-Stroh S, Barkham TMS, Nagarajan N, Sessions OM
EPI_ISL_477171	Department of Laboratory, Medicine Tan Tock Seng Hospital	Department of Laboratory, Medicine Tan Tock Seng Hospital	Chen YYC, Zair X, Li C, Tang WY, Maurer-Stroh S, Barkham TMS, Nagarajan N, Sessions OM
EPI_ISL_477172, EPI_ISL_477174, EPI_ISL_477175, EPI_ISL_477177, EPI_ISL_477178, EPI_ISL_477180, EPI_ISL_477182, EPI_ISL_477184, EPI_ISL_477187, EPI_ISL_477188, EPI_ISL_477189, EPI_ISL_477190, EPI_ISL_477191, EPI_ISL_477192, EPI_ISL_479482, EPI_ISL_479483, EPI_ISL_479484, EPI_ISL_479485, EPI_ISL_479486, EPI_ISL_479487, EPI_ISL_479488, EPI_ISL_479489, EPI_ISL_479490, EPI_ISL_479491, EPI_ISL_479492			
see above	Department of Laboratory Medicine Tan Tock Seng Hospital	Department of Laboratory Medicine Tan Tock Seng Hospital	Chen YYC, Zair X, Li C, Tang WY, Maurer-Stroh S, Barkham TMS, Nagarajan N, Sessions OM
EPI_ISL_479574, EPI_ISL_479575, EPI_ISL_479576, EPI_ISL_479577, EPI_ISL_479578, EPI_ISL_479579, EPI_ISL_479580, EPI_ISL_479581, EPI_ISL_479582, EPI_ISL_479583, EPI_ISL_479584, EPI_ISL_479585, EPI_ISL_479586, EPI_ISL_479587, EPI_ISL_479588, EPI_ISL_479589, EPI_ISL_479590, EPI_ISL_479591, EPI_ISL_479592, EPI_ISL_479593, EPI_ISL_479594, EPI_ISL_479595, EPI_ISL_479596, EPI_ISL_479597, EPI_ISL_479598, EPI_ISL_479599, EPI_ISL_479600, EPI_ISL_479601, EPI_ISL_479602, EPI_ISL_479603			
see above	National Public Health Laboratory, National Centre for Infectious Diseases	National Public Health Laboratory, National Centre for Infectious Diseases	Mak TM, Octavia S, Zhou Z, Chavatte JM, Cui L, Lin RTP
EPI_ISL_482672, EPI_ISL_482673, EPI_ISL_482674, EPI_ISL_482675, EPI_ISL_482676, EPI_ISL_482677, EPI_ISL_482678, EPI_ISL_482679, EPI_ISL_482680, EPI_ISL_482681, EPI_ISL_482682, EPI_ISL_482683, EPI_ISL_482684, EPI_ISL_482685, EPI_ISL_482686, EPI_ISL_482687, EPI_ISL_482688, EPI_ISL_482689, EPI_ISL_482690, EPI_ISL_482691, EPI_ISL_482692, EPI_ISL_482693, EPI_ISL_482694, EPI_ISL_482695, EPI_ISL_482696, EPI_ISL_482697, EPI_ISL_482698, EPI_ISL_482699			
see above	Singapore General Hospital	Department of Microbiology	Nurdyana Abdul Rahman, Kun Lee Lim, Chenhao Li, Kian Sing Chan, Lynette Oon, Kern Rei Chng, Niranjan Nagarajan, Karrie Ko
EPI_ISL_483576, EPI_ISL_483577, EPI_ISL_483578, EPI_ISL_483579, EPI_ISL_483580, EPI_ISL_483581, EPI_ISL_483582, EPI_ISL_483583, EPI_ISL_483584, EPI_ISL_483585, EPI_ISL_483586, EPI_ISL_483587, EPI_ISL_483588, EPI_ISL_483589, EPI_ISL_483590, EPI_ISL_483591, EPI_ISL_483592, EPI_ISL_483593, EPI_ISL_483594, EPI_ISL_483595, EPI_ISL_483596, EPI_ISL_483597, EPI_ISL_483598, EPI_ISL_483599, EPI_ISL_483600, EPI_ISL_483601, EPI_ISL_483602, EPI_ISL_483603, EPI_ISL_483604, EPI_ISL_483605, EPI_ISL_483606, EPI_ISL_483607, EPI_ISL_483608, EPI_ISL_483609, EPI_ISL_483610, EPI_ISL_483611, EPI_ISL_483612, EPI_ISL_483613, EPI_ISL_483614, EPI_ISL_483615, EPI_ISL_483616, EPI_ISL_483617, EPI_ISL_483618, EPI_ISL_483619, EPI_ISL_483620, EPI_ISL_483621, EPI_ISL_490049, EPI_ISL_490050, EPI_ISL_490051, EPI_ISL_490052, EPI_ISL_490053, EPI_ISL_490054, EPI_ISL_490055, EPI_ISL_490056, EPI_ISL_490057, EPI_ISL_490058, EPI_ISL_490059, EPI_ISL_490060, EPI_ISL_490061, EPI_ISL_490062, EPI_ISL_490063, EPI_ISL_490064, EPI_ISL_490065, EPI_ISL_490066, EPI_ISL_490067, EPI_ISL_490068, EPI_ISL_490069, EPI_ISL_490070, EPI_ISL_490071, EPI_ISL_490072, EPI_ISL_490073, EPI_ISL_490074, EPI_ISL_490075, EPI_ISL_490076, EPI_ISL_490077, EPI_ISL_490078, EPI_ISL_490079			
see above	National Public Health Laboratory, National Centre for Infectious Diseases	National Public Health Laboratory, National Centre for Infectious Diseases	Mak TM, Octavia S, Zhou Z, Chavatte JM, Cui L, Lin RTP
EPI_ISL_492978, EPI_ISL_492979	Department of Laboratory Medicine Tan Tock Seng Hospital	Department of Laboratory Medicine Tan Tock Seng Hospital	Chen YYC, Zair X, Li C, Tang WY, Maurer-Stroh S, Barkham TMS, Nagarajan N, Sessions OM
EPI_ISL_493390, EPI_ISL_493391, EPI_ISL_493392, EPI_ISL_493393, EPI_ISL_493394, EPI_ISL_493395, EPI_ISL_493396, EPI_ISL_493397, EPI_ISL_493398, EPI_ISL_493399, EPI_ISL_493400, EPI_ISL_493401, EPI_ISL_493402, EPI_ISL_493403, EPI_ISL_493404, EPI_ISL_493405, EPI_ISL_493406, EPI_ISL_493407, EPI_ISL_493408, EPI_ISL_493409, EPI_ISL_493410, EPI_ISL_493411, EPI_ISL_493412, EPI_ISL_493413, EPI_ISL_493414, EPI_ISL_493415, EPI_ISL_493416, EPI_ISL_493417, EPI_ISL_493418, EPI_ISL_493419, EPI_ISL_493420, EPI_ISL_493421, EPI_ISL_493422, EPI_ISL_493423, EPI_ISL_493424, EPI_ISL_493425, EPI_ISL_498564, EPI_ISL_498565, EPI_ISL_498566, EPI_ISL_498567, EPI_ISL_498568, EPI_ISL_498569, EPI_ISL_498570, EPI_ISL_498571, EPI_ISL_498572, EPI_ISL_498573, EPI_ISL_498574, EPI_ISL_498575, EPI_ISL_498576, EPI_ISL_498577, EPI_ISL_498578, EPI_ISL_498579, EPI_ISL_498580, EPI_ISL_498581, EPI_ISL_498582, EPI_ISL_498583, EPI_ISL_498584, EPI_ISL_498585, EPI_ISL_498586, EPI_ISL_498587, EPI_ISL_498588, EPI_ISL_498589, EPI_ISL_498590, EPI_ISL_498591, EPI_ISL_498592, EPI_ISL_498593, EPI_ISL_498594, EPI_ISL_498595, EPI_ISL_498596, EPI_ISL_498597, EPI_ISL_498598, EPI_ISL_498599, EPI_ISL_498600, EPI_ISL_498601, EPI_ISL_498602, EPI_ISL_498603, EPI_ISL_498604, EPI_ISL_498605, EPI_ISL_498606, EPI_ISL_498607, EPI_ISL_498608, EPI_ISL_498609, EPI_ISL_498610, EPI_ISL_498611, EPI_ISL_498612, EPI_ISL_498613, EPI_ISL_498614, EPI_ISL_498615, EPI_ISL_498616, EPI_ISL_498617, EPI_ISL_498618			
see above	National Public Health Laboratory, National Centre for Infectious Diseases	National Public Health Laboratory, National Centre for Infectious Diseases	Mak TM, Octavia S, Zhou Z, Chavatte JM, Cui L, Lin RTP
EPI_ISL_500539, EPI_ISL_500540, EPI_ISL_500541, EPI_ISL_500542, EPI_ISL_500543, EPI_ISL_500544, EPI_ISL_500545, EPI_ISL_500546, EPI_ISL_500547, EPI_ISL_500548, EPI_ISL_500549, EPI_ISL_500550, EPI_ISL_500551, EPI_ISL_500552, EPI_ISL_500553, EPI_ISL_500554, EPI_ISL_500555, EPI_ISL_500556, EPI_ISL_500557, EPI_ISL_500558, EPI_ISL_500559, EPI_ISL_500560, EPI_ISL_500561, EPI_ISL_500562, EPI_ISL_500563, EPI_ISL_500564, EPI_ISL_500565, EPI_ISL_500566, EPI_ISL_500567, EPI_ISL_500568, EPI_ISL_500569, EPI_ISL_500570, EPI_ISL_500571, EPI_ISL_500572			
see above	Singapore General Hospital	Department of Microbiology	Nurdyana Abdul Rahman, Kun Lee Lim, Chenhao Li, Kian Sing Chan, Lynette Oon, Kern Rei Chng, Niranjan Nagarajan, Karrie Ko
EPI_ISL_507002, EPI_ISL_507003, EPI_ISL_507004, EPI_ISL_507005, EPI_ISL_507006	Department of Laboratory Medicine, Tan Tock Seng Hospital	Department of Laboratory Medicine, Tan Tock Seng Hospital	Chen YYC, Zair X, Li C, Tang WY, Maurer-Stroh S, Barkham TMS, Nagarajan N, Sessions OM
EPI_ISL_509372, EPI_ISL_509373, EPI_ISL_509374, EPI_ISL_509375, EPI_ISL_509376, EPI_ISL_509377, EPI_ISL_509378, EPI_ISL_509379, EPI_ISL_509380, EPI_ISL_509381, EPI_ISL_509382, EPI_ISL_509383, EPI_ISL_509384, EPI_ISL_509385, EPI_ISL_509386, EPI_ISL_509387, EPI_ISL_509388, EPI_ISL_509389, EPI_ISL_509390, EPI_ISL_509391, EPI_ISL_509392, EPI_ISL_509393, EPI_ISL_509394, EPI_ISL_509395			
see above	Singapore General Hospital	Department of Microbiology	Nurdyana Abdul Rahman, Kun Lee Lim, Chenhao Li, Kian Sing Chan, Lynette Oon, Kern Rei Chng, Niranjan Nagarajan, Karrie Ko
EPI_ISL_512822, EPI_ISL_512823, EPI_ISL_512824, EPI_ISL_512825, EPI_ISL_512826, EPI_ISL_512827, EPI_ISL_512828, EPI_ISL_512829, EPI_ISL_512830, EPI_ISL_512831, EPI_ISL_512832, EPI_ISL_512833, EPI_ISL_512834, EPI_ISL_512835, EPI_ISL_512836, EPI_ISL_512837, EPI_ISL_512838, EPI_ISL_512839, EPI_ISL_512840, EPI_ISL_512841, EPI_ISL_512842			
see above	National Public Health Laboratory, National Centre for Infectious Diseases	National Public Health Laboratory, National Centre for Infectious Diseases	Mak TM, Octavia S, Zhou Z, Chavatte JM, Cui L, Lin RTP
EPI_ISL_516801, EPI_ISL_516802, EPI_ISL_516803, EPI_ISL_516804, EPI_ISL_516805	Department of Laboratory Medicine, Tan Tock Seng Hospital	Department of Laboratory Medicine, Tan Tock Seng Hospital	Chen YYC, Zair X, Li C, Tang WY, Maurer-Stroh S, Barkham TMS, Nagarajan N, Sessions OM
EPI_ISL_516807, EPI_ISL_516808, EPI_ISL_516809, EPI_ISL_516810, EPI_ISL_516811, EPI_ISL_516812, EPI_ISL_516813, EPI_ISL_516814, EPI_ISL_516815, EPI_ISL_516816, EPI_ISL_516817, EPI_ISL_516818, EPI_ISL_516819, EPI_ISL_516820, EPI_ISL_516821, EPI_ISL_516822, EPI_ISL_516823, EPI_ISL_516824, EPI_ISL_516825, EPI_ISL_516826, EPI_ISL_516827, EPI_ISL_516828			
see above	National Public Health Laboratory, National Centre for Infectious Diseases	National Public Health Laboratory, National Centre for Infectious Diseases	Mak TM, Octavia S, Zhou Z, Cui L, Lin RTP
EPI_ISL_517996, EPI_ISL_517997, EPI_ISL_517998, EPI_ISL_517999, EPI_ISL_518000, EPI_ISL_518001, EPI_ISL_518002, EPI_ISL_518003, EPI_ISL_518004, EPI_ISL_518005, EPI_ISL_518006, EPI_ISL_518007, EPI_ISL_518008, EPI_ISL_518009, EPI_ISL_518010, EPI_ISL_518011, EPI_ISL_518012, EPI_ISL_518013, EPI_ISL_518014, EPI_ISL_518015, EPI_ISL_518016, EPI_ISL_518017, EPI_ISL_524445, EPI_ISL_524446, EPI_ISL_524447, EPI_ISL_524448, EPI_ISL_524449			
see above	Singapore General Hospital	Department of Microbiology	Nurdyana Abdul Rahman, Kun Lee Lim, Chenhao Li, Kian Sing Chan, Lynette Oon, Kern Rei Chng, Niranjan Nagarajan, Karrie Ko
EPI_ISL_527359, EPI_ISL_527360, EPI_ISL_527361, EPI_ISL_527362, EPI_ISL_527363, EPI_ISL_527364, EPI_ISL_527365, EPI_ISL_527366, EPI_ISL_527367, EPI_ISL_527368, EPI_ISL_527369, EPI_ISL_527370, EPI_ISL_527371, EPI_ISL_527372, EPI_ISL_527373, EPI_ISL_527374, EPI_ISL_527375, EPI_ISL_527376, EPI_ISL_527377, EPI_ISL_527378, EPI_ISL_527379, EPI_ISL_536412, EPI_ISL_536413, EPI_ISL_536414, EPI_ISL_536415, EPI_ISL_536416, EPI_ISL_536417, EPI_ISL_536418, EPI_ISL_536419, EPI_ISL_536420, EPI_ISL_536421, EPI_ISL_536422, EPI_ISL_536423, EPI_ISL_536424, EPI_ISL_536425, EPI_ISL_536426, EPI_ISL_536427, EPI_ISL_536428, EPI_ISL_536429, EPI_ISL_536430, EPI_ISL_536431, EPI_ISL_536432, EPI_ISL_536433, EPI_ISL_536434, EPI_ISL_536435, EPI_ISL_536436, EPI_ISL_536437, EPI_ISL_536438, EPI_ISL_536439, EPI_ISL_536440, EPI_ISL_536441, EPI_ISL_536442, EPI_ISL_536443, EPI_ISL_536444, EPI_ISL_536445, EPI_ISL_536446, EPI_ISL_536447, EPI_ISL_536448, EPI_ISL_536449, EPI_ISL_536450, EPI_ISL_536451, EPI_ISL_536452, EPI_ISL_536453, EPI_ISL_536454			
see above	National Public Health Laboratory, National Centre for Infectious Diseases	National Public Health Laboratory, National Centre for Infectious Diseases	Mak TM, Octavia S, Zhou Z, Cui L, Lin RTP
EPI_ISL_538435, EPI_ISL_538436, EPI_ISL_538437, EPI_ISL_538438, EPI_ISL_538439, EPI_ISL_538440, EPI_ISL_538441, EPI_ISL_538442, EPI_ISL_538443, EPI_ISL_538444, EPI_ISL_538445, EPI_ISL_538446, EPI_ISL_538447, EPI_ISL_538448, EPI_ISL_538449, EPI_ISL_538450, EPI_ISL_538451, EPI_ISL_538452, EPI_ISL_538453, EPI_ISL_538454, EPI_ISL_538455, EPI_ISL_538456, EPI_ISL_538457, EPI_ISL_538458, EPI_ISL_538459, EPI_ISL_538460, EPI_ISL_538461, EPI_ISL_538462, EPI_ISL_538463, EPI_ISL_538464, EPI_ISL_538465, EPI_ISL_538466, EPI_ISL_538467, EPI_ISL_538468, EPI_ISL_538469, EPI_ISL_538470, EPI_ISL_538471, EPI_ISL_538472, EPI_ISL_538473, EPI_ISL_538474, EPI_ISL_538475, EPI_ISL_538476, EPI_ISL_538477, EPI_ISL_538478, EPI_ISL_538479, EPI_ISL_538480, EPI_ISL_538481, EPI_ISL_538482, EPI_ISL_538483, EPI_ISL_538484, EPI_ISL_538485, EPI_ISL_538486, EPI_ISL_538487, EPI_ISL_538488, EPI_ISL_538489, EPI_ISL_538490, EPI_ISL_538491, EPI_ISL_538492, EPI_ISL_538493, EPI_ISL_538494, EPI_ISL_538495, EPI_ISL_538496, EPI_ISL_538497			
see above	Department of Laboratory Medicine, Tan Tock Seng Hospital	Department of Laboratory Medicine, Tan Tock Seng Hospital	Chen YYC, Zair X, Lim JX, Li C, Tang WY, Maurer-Stroh S, Barkham TMS, Nagarajan N, Sessions OM
EPI_ISL_548972, EPI_ISL_548973, EPI_ISL_548974, EPI_ISL_548975, EPI_ISL_548976, EPI_ISL_548977, EPI_ISL_548978, EPI_ISL_548979, EPI_ISL_548980, EPI_ISL_548981, EPI_ISL_548982, EPI_ISL_548983, EPI_ISL_548984, EPI_ISL_548985, EPI_ISL_548986, EPI_ISL_548987, EPI_ISL_548988, EPI_ISL_548989, EPI_ISL_548990, EPI_ISL_548991, EPI_ISL_548992, EPI_ISL_548993, EPI_ISL_548994, EPI_ISL_548995, EPI_ISL_548996, EPI_ISL_548997, EPI_ISL_548998, EPI_ISL_548999, EPI_ISL_549000, EPI_ISL_549001, EPI_ISL_549002, EPI_ISL_549003, EPI_ISL_549004, EPI_ISL_549005			
see above	National Public Health Laboratory, National Centre for Infectious Diseases	National Public Health Laboratory, National Centre for Infectious Diseases	Mak TM, Octavia S, Zhou Z, Cui L, Lin RTP
EPI_ISL_574479, EPI_ISL_574480, EPI_ISL_574481, EPI_ISL_574482, EPI_ISL_574483, EPI_ISL_574484, EPI_ISL_574485, EPI_ISL_574486, EPI_ISL_574487, EPI_ISL_574488, EPI_ISL_574489, EPI_ISL_574490, EPI_ISL_574491, EPI_ISL_574492			
see above	Programme in Emerging Infectious Diseases, Duke-NUS Medical School	National Public Health Laboratory, National Centre for Infectious Diseases	Tze Minn Mak, Sophie Octavia, Zhenyang Zhou, Danielle E Anderson, Adrian Eng Zheng Kang, Lin Cui, Raymond Tzer Pin Lin
EPI_ISL_574493, EPI_ISL_574494, EPI_ISL_574495, EPI_ISL_574496, EPI_ISL_574497, EPI_ISL_574498, EPI_ISL_574499, EPI_ISL_574500, EPI_ISL_574501, EPI_ISL_574502, EPI_ISL_574503, EPI_ISL_574504, EPI_ISL_574505, EPI_ISL_574506, EPI_ISL_574507, EPI_ISL_574508, EPI_ISL_574509, EPI_ISL_574510,			

EPI_ISL_574511, EPI_ISL_574512, EPI_ISL_574513, EPI_ISL_574514, EPI_ISL_574515, EPI_ISL_574516, EPI_ISL_574517, EPI_ISL_574518, EPI_ISL_574519, EPI_ISL_574520, EPI_ISL_574521, EPI_ISL_574522, EPI_ISL_574523, EPI_ISL_574524, EPI_ISL_574525, EPI_ISL_574526, EPI_ISL_574527, EPI_ISL_574528, EPI_ISL_574529, EPI_ISL_574530, EPI_ISL_574531, EPI_ISL_574532, EPI_ISL_574533, EPI_ISL_574534, EPI_ISL_574535, EPI_ISL_574536, EPI_ISL_574537, EPI_ISL_574538, EPI_ISL_574539, EPI_ISL_574540, EPI_ISL_574541, EPI_ISL_574542			
see above	National Public Health Laboratory, National Centre for Infectious Diseases	National Public Health Laboratory, National Centre for Infectious Diseases	Tze Minn Mak, Sophie Octavia, Zhenyang Zhou, Lin Cui, Raymond Tzer Pin Lin
EPI_ISL_583893, EPI_ISL_583894, EPI_ISL_583895, EPI_ISL_583896, EPI_ISL_583897, EPI_ISL_583898, EPI_ISL_583899, EPI_ISL_583900	Singapore General Hospital	Department of Microbiology	Nurdyana Abdul Rahman, Kun Lee Lim, Chenhao Li, Sui Sin Goh, Kenneth Xin Long Chan, Kian Sing Chan, Lynette Oon, Kern Rei Chng, Niranjan Nagarajan, Karrie Ko
EPI_ISL_596456, EPI_ISL_596457, EPI_ISL_596458, EPI_ISL_596459, EPI_ISL_596460, EPI_ISL_596461, EPI_ISL_596462, EPI_ISL_596463, EPI_ISL_596464, EPI_ISL_596465, EPI_ISL_596466, EPI_ISL_596467, EPI_ISL_596468, EPI_ISL_596469, EPI_ISL_596470, EPI_ISL_596471, EPI_ISL_596472, EPI_ISL_596473, EPI_ISL_596474, EPI_ISL_596475, EPI_ISL_596476, EPI_ISL_596477, EPI_ISL_596478, EPI_ISL_596479, EPI_ISL_596480, EPI_ISL_596481, EPI_ISL_596482, EPI_ISL_596483, EPI_ISL_596484, EPI_ISL_596485, EPI_ISL_596486, EPI_ISL_596487, EPI_ISL_596488, EPI_ISL_596489, EPI_ISL_596490, EPI_ISL_596491, EPI_ISL_596492, EPI_ISL_596493, EPI_ISL_596494, EPI_ISL_596495, EPI_ISL_596496, EPI_ISL_596497, EPI_ISL_605817, EPI_ISL_605818, EPI_ISL_605819, EPI_ISL_605820, EPI_ISL_605821, EPI_ISL_605822, EPI_ISL_605823, EPI_ISL_605824			
see above	National Public Health Laboratory, National Centre for Infectious Diseases	National Public Health Laboratory, National Centre for Infectious Diseases	Tze Minn Mak, Sophie Octavia, Zhenyang Zhou, Lin Cui, Raymond Tzer Pin Lin
EPI_ISL_610152, EPI_ISL_610153, EPI_ISL_610154	Singapore General Hospital	Department of Microbiology	Nurdyana Abdul Rahman, Kun Lee Lim, Chenhao Li, Sui Sin Goh, Kenneth Xin Long Chan, Kian Sing Chan, Lynette Oon, Kern Rei Chng, Niranjan Nagarajan, Karrie Ko
EPI_ISL_626628, EPI_ISL_626629, EPI_ISL_626630, EPI_ISL_626631, EPI_ISL_626632, EPI_ISL_626633, EPI_ISL_626634, EPI_ISL_626635, EPI_ISL_626636, EPI_ISL_626637, EPI_ISL_626638, EPI_ISL_626639, EPI_ISL_626640, EPI_ISL_626641, EPI_ISL_626642, EPI_ISL_626643, EPI_ISL_626644, EPI_ISL_626645, EPI_ISL_626646, EPI_ISL_645115, EPI_ISL_645116, EPI_ISL_645117, EPI_ISL_645118, EPI_ISL_645119, EPI_ISL_645120, EPI_ISL_645121, EPI_ISL_645122, EPI_ISL_645123, EPI_ISL_645124, EPI_ISL_645125, EPI_ISL_645126, EPI_ISL_645127, EPI_ISL_645128, EPI_ISL_645129, EPI_ISL_645130, EPI_ISL_645131, EPI_ISL_645132			
see above	National Public Health Laboratory, National Centre for Infectious Diseases	National Public Health Laboratory, National Centre for Infectious Diseases	Tze Minn Mak, Sophie Octavia, Zhenyang Zhou, Lin Cui, Raymond Tzer Pin Lin
EPI_ISL_648043, EPI_ISL_648044, EPI_ISL_648045, EPI_ISL_648046, EPI_ISL_648047, EPI_ISL_648048, EPI_ISL_648049, EPI_ISL_648050, EPI_ISL_648051, EPI_ISL_648052, EPI_ISL_648053, EPI_ISL_648054, EPI_ISL_648055, EPI_ISL_648056, EPI_ISL_648057, EPI_ISL_648058, EPI_ISL_648059, EPI_ISL_648060, EPI_ISL_648061, EPI_ISL_648062, EPI_ISL_648063, EPI_ISL_648064, EPI_ISL_648065, EPI_ISL_648066, EPI_ISL_648067, EPI_ISL_648068, EPI_ISL_648069, EPI_ISL_648070, EPI_ISL_648071, EPI_ISL_648072, EPI_ISL_648073, EPI_ISL_648074, EPI_ISL_648075, EPI_ISL_648076, EPI_ISL_648077, EPI_ISL_648078, EPI_ISL_648079, EPI_ISL_648080, EPI_ISL_648081, EPI_ISL_648082, EPI_ISL_648083, EPI_ISL_648084, EPI_ISL_648085, EPI_ISL_648086, EPI_ISL_648087, EPI_ISL_648088, EPI_ISL_648089, EPI_ISL_648090, EPI_ISL_648091, EPI_ISL_648092, EPI_ISL_648093, EPI_ISL_648094, EPI_ISL_648095, EPI_ISL_648096, EPI_ISL_648097, EPI_ISL_648098, EPI_ISL_648099, EPI_ISL_648100, EPI_ISL_648101, EPI_ISL_648102, EPI_ISL_648103, EPI_ISL_648104, EPI_ISL_648105, EPI_ISL_648106, EPI_ISL_648107, EPI_ISL_648108, EPI_ISL_648109, EPI_ISL_648110, EPI_ISL_648111, EPI_ISL_648112, EPI_ISL_648113, EPI_ISL_648114, EPI_ISL_648115, EPI_ISL_648116, EPI_ISL_648117, EPI_ISL_648118, EPI_ISL_648119, EPI_ISL_648120, EPI_ISL_648121, EPI_ISL_648610, EPI_ISL_648611, EPI_ISL_648612, EPI_ISL_648613, EPI_ISL_648614, EPI_ISL_648615, EPI_ISL_648616, EPI_ISL_648617, EPI_ISL_648618, EPI_ISL_648619, EPI_ISL_648620, EPI_ISL_648621, EPI_ISL_648622, EPI_ISL_648623, EPI_ISL_648624, EPI_ISL_648625, EPI_ISL_648626, EPI_ISL_648627, EPI_ISL_648628, EPI_ISL_648629, EPI_ISL_648630, EPI_ISL_648631, EPI_ISL_648632, EPI_ISL_648633, EPI_ISL_648634, EPI_ISL_648635, EPI_ISL_648636, EPI_ISL_648637, EPI_ISL_648638, EPI_ISL_648639, EPI_ISL_648640, EPI_ISL_648641, EPI_ISL_648642, EPI_ISL_648643, EPI_ISL_648644, EPI_ISL_648645, EPI_ISL_648646, EPI_ISL_648647, EPI_ISL_648648, EPI_ISL_648649, EPI_ISL_648650, EPI_ISL_648651, EPI_ISL_648652, EPI_ISL_648653, EPI_ISL_648654, EPI_ISL_648655, EPI_ISL_648656, EPI_ISL_648657, EPI_ISL_648658, EPI_ISL_648659, EPI_ISL_648660, EPI_ISL_648661, EPI_ISL_648662, EPI_ISL_648663, EPI_ISL_648664, EPI_ISL_648665, EPI_ISL_648666, EPI_ISL_648667, EPI_ISL_648668, EPI_ISL_648669, EPI_ISL_648670, EPI_ISL_648671, EPI_ISL_648672, EPI_ISL_648673, EPI_ISL_648674, EPI_ISL_648675, EPI_ISL_648676, EPI_ISL_648678, EPI_ISL_648679, EPI_ISL_648680, EPI_ISL_648681, EPI_ISL_648682, EPI_ISL_648683, EPI_ISL_648684, EPI_ISL_648685, EPI_ISL_648686, EPI_ISL_648687, EPI_ISL_648688, EPI_ISL_648689, EPI_ISL_648690, EPI_ISL_648691, EPI_ISL_648692, EPI_ISL_648693, EPI_ISL_648694, EPI_ISL_648695, EPI_ISL_648696, EPI_ISL_648697, EPI_ISL_648698, EPI_ISL_648699, EPI_ISL_648700, EPI_ISL_648701, EPI_ISL_648702, EPI_ISL_648703, EPI_ISL_648704, EPI_ISL_648705, EPI_ISL_648706, EPI_ISL_648707, EPI_ISL_648708, EPI_ISL_648709, EPI_ISL_648710, EPI_ISL_648711, EPI_ISL_648712, EPI_ISL_648713, EPI_ISL_648714, EPI_ISL_648715, EPI_ISL_648716, EPI_ISL_648717, EPI_ISL_648718, EPI_ISL_648719, EPI_ISL_648720, EPI_ISL_648721, EPI_ISL_648722, EPI_ISL_648723, EPI_ISL_648724, EPI_ISL_648725, EPI_ISL_648726, EPI_ISL_648727, EPI_ISL_648728, EPI_ISL_648729, EPI_ISL_648730, EPI_ISL_648731, EPI_ISL_648732, EPI_ISL_648733, EPI_ISL_648734, EPI_ISL_648735, EPI_ISL_648736, EPI_ISL_648737, EPI_ISL_648738, EPI_ISL_648739, EPI_ISL_648740, EPI_ISL_648741, EPI_ISL_648742, EPI_ISL_648743, EPI_ISL_648744, EPI_ISL_648745, EPI_ISL_648746, EPI_ISL_648747, EPI_ISL_648748, EPI_ISL_648749, EPI_ISL_648750, EPI_ISL_648751, EPI_ISL_648752, EPI_ISL_648753, EPI_ISL_648754, EPI_ISL_648755, EPI_ISL_648756, EPI_ISL_648757, EPI_ISL_648758, EPI_ISL_648759, EPI_ISL_648760, EPI_ISL_648761, EPI_ISL_648762, EPI_ISL_648763, EPI_ISL_648764, EPI_ISL_648765, EPI_ISL_648766, EPI_ISL_648767, EPI_ISL_648768, EPI_ISL_648769, EPI_ISL_648770, EPI_ISL_648771, EPI_ISL_648772, EPI_ISL_648773, EPI_ISL_648774, EPI_ISL_648775, EPI_ISL_648776, EPI_ISL_648777, EPI_ISL_648778, EPI_ISL_648779, EPI_ISL_648780, EPI_ISL_648781, EPI_ISL_648782, EPI_ISL_648783, EPI_ISL_648784, EPI_ISL_648785, EPI_ISL_648786, EPI_ISL_648787, EPI_ISL_648788, EPI_ISL_648789, EPI_ISL_648790, EPI_ISL_648791, EPI_ISL_648792, EPI_ISL_648793, EPI_ISL_648794, EPI_ISL_648795, EPI_ISL_648796, EPI_ISL_648797, EPI_ISL_648798, EPI_ISL_648799, EPI_ISL_648800, EPI_ISL_648801, EPI_ISL_648802, EPI_ISL_648803, EPI_ISL_648804, EPI_ISL_648805, EPI_ISL_648806, EPI_ISL_648807, EPI_ISL_648808, EPI_ISL_648809, EPI_ISL_648810, EPI_ISL_648811, EPI_ISL_648812, EPI_ISL_648813, EPI_ISL_648814, EPI_ISL_648815, EPI_ISL_648816, EPI_ISL_648817, EPI_ISL_648818, EPI_ISL_648819, EPI_ISL_648820			
see above	Department of Laboratory Medicine, Tan Tock Seng Hospital	Department of Laboratory Medicine, Tan Tock Seng Hospital	Chen YYC, Zair X, Lim JX, Li C, Tang WY, Maurer-Stroh S, Barkham TMS, Nagarajan N, Sessions OM
EPI_ISL_654810, EPI_ISL_654811, EPI_ISL_654812, EPI_ISL_654813, EPI_ISL_654814, EPI_ISL_654815, EPI_ISL_654816, EPI_ISL_654817, EPI_ISL_693307, EPI_ISL_693308, EPI_ISL_693309, EPI_ISL_693310, EPI_ISL_693311, EPI_ISL_693312, EPI_ISL_693313, EPI_ISL_693314, EPI_ISL_693315, EPI_ISL_693316, EPI_ISL_693317, EPI_ISL_693318, EPI_ISL_693319, EPI_ISL_693320, EPI_ISL_693321, EPI_ISL_693322, EPI_ISL_693323, EPI_ISL_693324, EPI_ISL_693325, EPI_ISL_693326, EPI_ISL_693327, EPI_ISL_693328, EPI_ISL_693329, EPI_ISL_693330, EPI_ISL_693331, EPI_ISL_728179, EPI_ISL_728180, EPI_ISL_728181, EPI_ISL_728182, EPI_ISL_728183, EPI_ISL_728184, EPI_ISL_728185, EPI_ISL_728186, EPI_ISL_728187, EPI_ISL_728188, EPI_ISL_728189, EPI_ISL_728190, EPI_ISL_728191, EPI_ISL_728192, EPI_ISL_728193, EPI_ISL_728194, EPI_ISL_728195, EPI_ISL_728196, EPI_ISL_728197, EPI_ISL_728198, EPI_ISL_728199, EPI_ISL_728200, EPI_ISL_728201, EPI_ISL_754074, EPI_ISL_754075, EPI_ISL_754076, EPI_ISL_754077, EPI_ISL_754078, EPI_ISL_754079, EPI_ISL_754080, EPI_ISL_754081, EPI_ISL_754082, EPI_ISL_754083, EPI_ISL_754084, EPI_ISL_754085, EPI_ISL_754086, EPI_ISL_754087, EPI_ISL_754088, EPI_ISL_754089, EPI_ISL_754090, EPI_ISL_754091, EPI_ISL_754092, EPI_ISL_754093, EPI_ISL_754094, EPI_ISL_754095, EPI_ISL_754096, EPI_ISL_754097, EPI_ISL_754098, EPI_ISL_754099, EPI_ISL_754100, EPI_ISL_754101, EPI_ISL_754102, EPI_ISL_754103, EPI_ISL_754104, EPI_ISL_754105, EPI_ISL_754106, EPI_ISL_754107, EPI_ISL_754108			
see above	National Public Health Laboratory, National Centre for Infectious Diseases	National Public Health Laboratory, National Centre for Infectious Diseases	Tze Minn Mak, Sophie Octavia, Zhenyang Zhou, Lin Cui, Raymond Tzer Pin Lin
EPI_ISL_768324	Singapore General Hospital	Department of Microbiology	Nurdyana Abdul Rahman, Kun Lee Lim, Chenhao Li, Sui Sin Goh, Kenneth Xin Long Chan, Kian Sing Chan, Lynette Oon, Kern Rei Chng, Niranjan Nagarajan, Karrie Ko
EPI_ISL_768618, EPI_ISL_768619, EPI_ISL_768620, EPI_ISL_768621, EPI_ISL_768622, EPI_ISL_768623, EPI_ISL_768624, EPI_ISL_768625, EPI_ISL_768626, EPI_ISL_768627, EPI_ISL_768628, EPI_ISL_768629, EPI_ISL_768630, EPI_ISL_768631, EPI_ISL_768632, EPI_ISL_803958, EPI_ISL_803959, EPI_ISL_803960, EPI_ISL_803961, EPI_ISL_803962, EPI_ISL_803963, EPI_ISL_803964, EPI_ISL_803965, EPI_ISL_803966, EPI_ISL_803967, EPI_ISL_803968, EPI_ISL_803969, EPI_ISL_803970, EPI_ISL_803971, EPI_ISL_803972, EPI_ISL_803973, EPI_ISL_803974, EPI_ISL_803975, EPI_ISL_803976, EPI_ISL_803977, EPI_ISL_803978, EPI_ISL_803979, EPI_ISL_803980, EPI_ISL_803981, EPI_ISL_803982, EPI_ISL_803983, EPI_ISL_803984, EPI_ISL_803985, EPI_ISL_803986, EPI_ISL_803987, EPI_ISL_803988, EPI_ISL_803989, EPI_ISL_803990, EPI_ISL_803991, EPI_ISL_803992, EPI_ISL_803993, EPI_ISL_803994, EPI_ISL_803995, EPI_ISL_803996, EPI_ISL_803997, EPI_ISL_803998, EPI_ISL_803999, EPI_ISL_804000, EPI_ISL_804001, EPI_ISL_804002, EPI_ISL_804003, EPI_ISL_804004, EPI_ISL_804005, EPI_ISL_804006, EPI_ISL_825064, EPI_ISL_825065, EPI_ISL_825066, EPI_ISL_825067, EPI_ISL_825068, EPI_ISL_825069, EPI_ISL_825070, EPI_ISL_825071, EPI_ISL_825072, EPI_ISL_825073, EPI_ISL_825074, EPI_ISL_825075, EPI_ISL_825076, EPI_ISL_825077, EPI_ISL_825078, EPI_ISL_825079, EPI_ISL_825080, EPI_ISL_825081, EPI_ISL_825082, EPI_ISL_825083, EPI_ISL_833373, EPI_ISL_833374, EPI_ISL_833375, EPI_ISL_833376, EPI_ISL_833377, EPI_ISL_833378, EPI_ISL_833379, EPI_ISL_833380, EPI_ISL_833381, EPI_ISL_833382, EPI_ISL_833383, EPI_ISL_833384, EPI_ISL_833385, EPI_ISL_833386, EPI_ISL_833387, EPI_ISL_857469, EPI_ISL_857470, EPI_ISL_857471, EPI_ISL_857472, EPI_ISL_857473, EPI_ISL_857474, EPI_ISL_857475, EPI_ISL_857476, EPI_ISL_857477, EPI_ISL_857478, EPI_ISL_857479, EPI_ISL_857480, EPI_ISL_857481, EPI_ISL_857482, EPI_ISL_857483, EPI_ISL_857484, EPI_ISL_857485			
see above	National Public Health Laboratory, National Centre for Infectious Diseases	National Public Health Laboratory, National Centre for Infectious Diseases	Tze Minn Mak, Sophie Octavia, Zhenyang Zhou, Lin Cui, Raymond Tzer Pin Lin
EPI_ISL_862816, EPI_ISL_862817, EPI_ISL_862818, EPI_ISL_862819, EPI_ISL_862820, EPI_ISL_862821, EPI_ISL_862822, EPI_ISL_862823, EPI_ISL_862824, EPI_ISL_862825, EPI_ISL_862826, EPI_ISL_862827, EPI_ISL_862828, EPI_ISL_862829, EPI_ISL_862830, EPI_ISL_891231, EPI_ISL_891232, EPI_ISL_891233, EPI_ISL_891234, EPI_ISL_891235, EPI_ISL_891236, EPI_ISL_891237, EPI_ISL_891238, EPI_ISL_891239, EPI_ISL_891240, EPI_ISL_891241, EPI_ISL_891242, EPI_ISL_891243, EPI_ISL_891244, EPI_ISL_891245, EPI_ISL_891246, EPI_ISL_891247, EPI_ISL_891248			
see above	National Public Health Laboratory, National Centre for Infectious Diseases	National Public Health Laboratory, National Centre for Infectious Diseases	Tze Minn Mak, Zhenyang Zhou, Lin Cui, Raymond Tzer Pin Lin
EPI_ISL_891251, EPI_ISL_891252, EPI_ISL_891253	Singapore General Hospital	Department of Microbiology	Nurdyana Abdul Rahman, Kun Lee Lim, Chenhao Li, Sui Sin Goh, Kenneth Xin Long Chan, Kian Sing Chan, Lynette Oon, Kern Rei Chng, Niranjan Nagarajan, Karrie Ko
EPI_ISL_906833, EPI_ISL_906834, EPI_ISL_906835, EPI_ISL_906836, EPI_ISL_906837, EPI_ISL_906838, EPI_ISL_906839, EPI_ISL_906840, EPI_ISL_906841, EPI_ISL_906842, EPI_ISL_906843, EPI_ISL_906844, EPI_ISL_906845, EPI_ISL_906846, EPI_ISL_937515, EPI_ISL_937516, EPI_ISL_937517, EPI_ISL_937518, EPI_ISL_937519, EPI_ISL_937520, EPI_ISL_937521, EPI_ISL_937522, EPI_ISL_937523, EPI_ISL_937524, EPI_ISL_937525, EPI_ISL_937526, EPI_ISL_937527, EPI_ISL_937528, EPI_ISL_937529, EPI_ISL_937530, EPI_ISL_953385, EPI_ISL_953386, EPI_ISL_953387, EPI_ISL_953388, EPI_ISL_953389, EPI_ISL_953390, EPI_ISL_953391, EPI_ISL_953392, EPI_ISL_953393, EPI_ISL_953394, EPI_ISL_953395, EPI_ISL_953396, EPI_ISL_953397, EPI_ISL_953398, EPI_ISL_953399, EPI_ISL_953400, EPI_ISL_953401, EPI_ISL_981007, EPI_ISL_981008, EPI_ISL_981009, EPI_ISL_981010, EPI_ISL_981011, EPI_ISL_981012, EPI_ISL_981013, EPI_ISL_981014, EPI_ISL_981015, EPI_ISL_981016, EPI_ISL_981017, EPI_ISL_981018, EPI_ISL_981019, EPI_ISL_981020, EPI_ISL_981021, EPI_ISL_981022, EPI_ISL_981023, EPI_ISL_981024, EPI_ISL_981025, EPI_ISL_981026, EPI_ISL_981027, EPI_ISL_981028, EPI_ISL_981029, EPI_ISL_995294, EPI_ISL_995295, EPI_ISL_995297, EPI_ISL_995298, EPI_ISL_995299, EPI_ISL_995300, EPI_ISL_995301, EPI_ISL_995302, EPI_ISL_995303, EPI_ISL_995305, EPI_ISL_995306, EPI_ISL_995307			
see above	National Public Health Laboratory, National Centre for Infectious Diseases	National Public Health Laboratory, National Centre for Infectious Diseases	Tze Minn Mak, Zhenyang Zhou, Lin Cui, Raymond Tzer Pin Lin
