# Supplementary material for: CalmBelt: Rapid SARS-CoV-2 Genome Characterization for Outbreak Tracking
Source: Front Med (Lausanne). 2021 Dec 14;8:790662. doi: 10.3389/fmed.2021.790662 (PMC8712659; doi:10.3389/fmed.2021.790662)
Supplement: Supplementary Figure 1 — Increasing mutation rate of 3,406 genomes from Singapore downloaded from GISAID (Jan 2020–June 2021). Y-axis represents an average number of mutations per week. [file Data_Sheet_1.PDF]

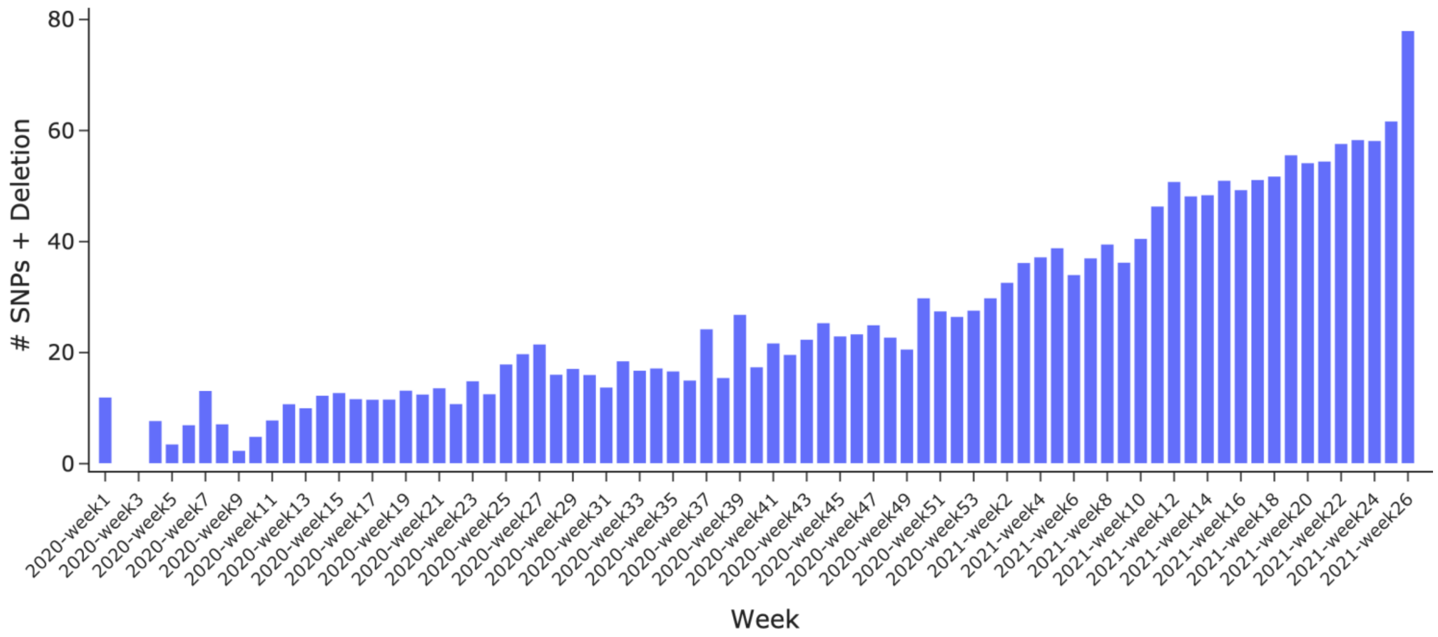

Supplementary figure 1. Increasing mutation rate of 3,406 genomes from Singapore downloaded from GISAID (Jan 2020 - June 2021). Y-axis represents an average number of mutations per week.

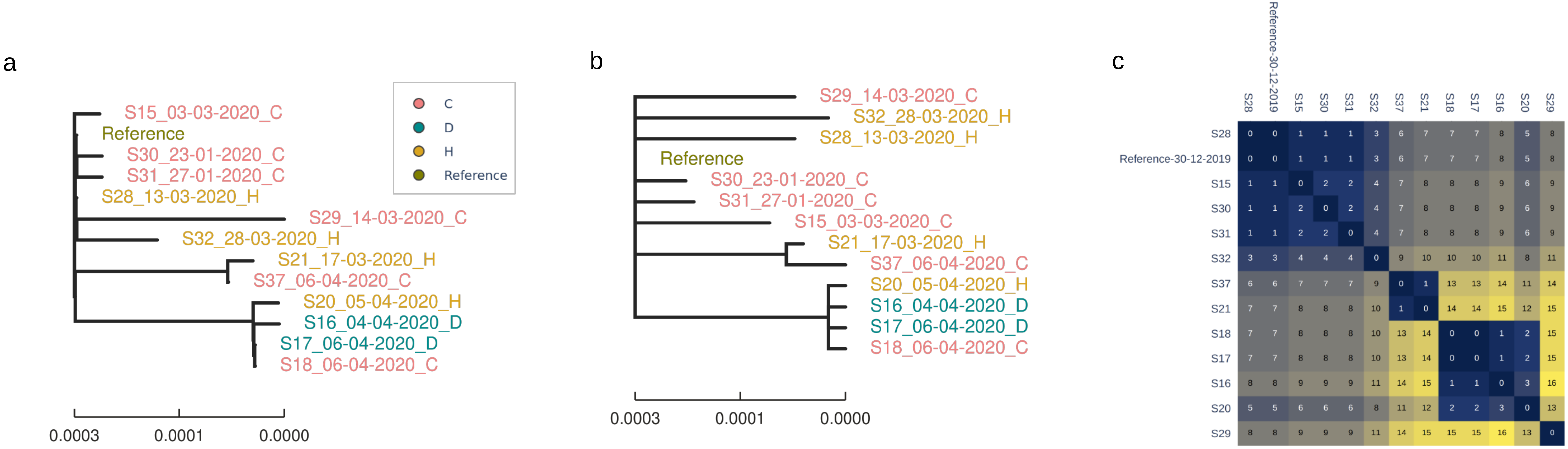

Supplementary figure 2. In addition to a similarity tree and a list of mutations in Figure 1b, CalmBelt provides a phylogenetic tree (a; IQTREE2 used in Pangolin), a transmission tree (b; TreeTime used in Nextstrain), and a heatmap (c) capturing the number of different nucleotides between every pair of genomes.

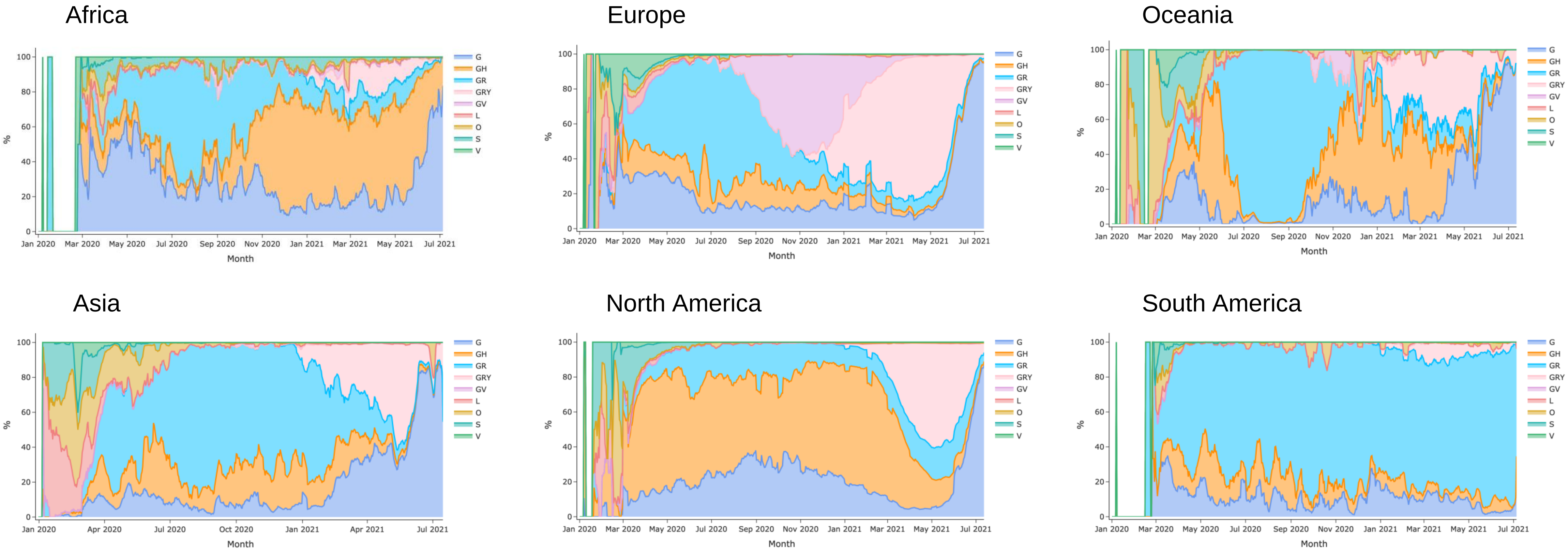

Supplementary figure 3. Time-series plots show the trend of GISAID clades across continents. We note that the trends might be slightly different from Nextstrain due to the subsampling process used in Nextstrain.

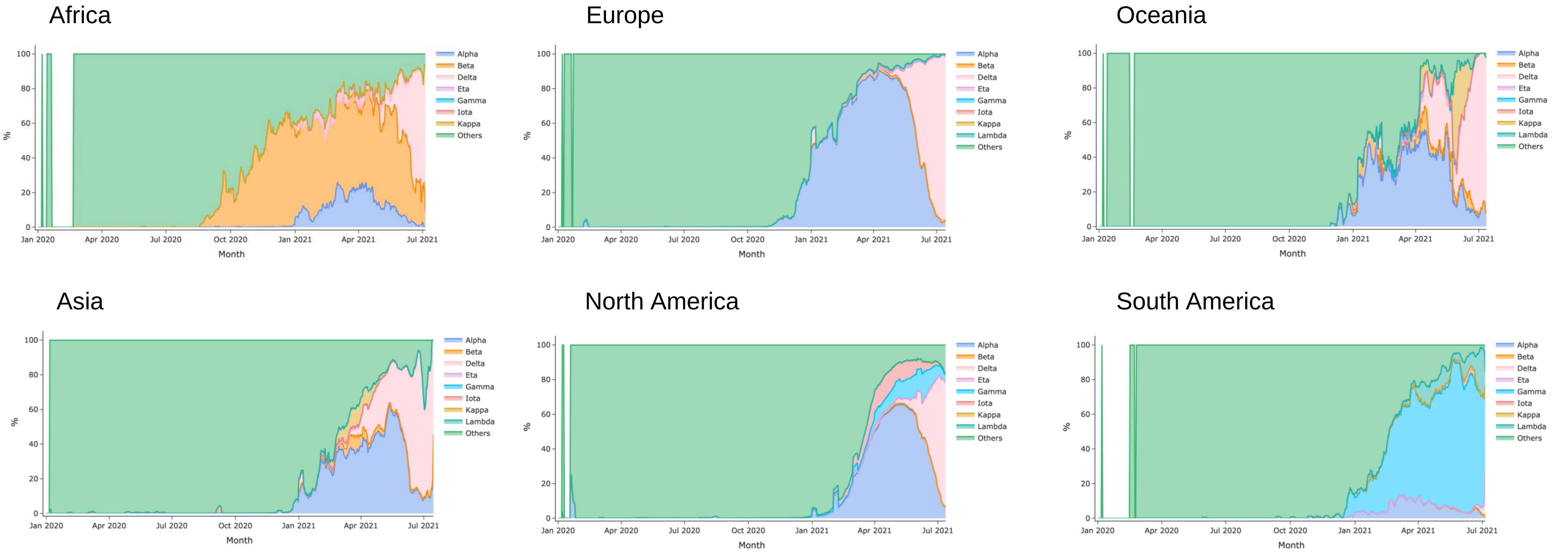

Supplementary figure 4. Time-series plots show the trend of PANGO lineage (based on the greek alphabets) across continents.

Mar 2020

Country	Percentage	# B.6.6 genomes	# total genomes	Lineage
Cambodia	10.53	2	19	B.6.6
Singapore	9.45	36	381	B.6.6
Malaysia	8.93	5	56	B.6.6
India	5.18	13	251	B.6.6
Australia	0.22	5	2254	B.6.6

April 2020

Country	Percentage	# B.6.6 genomes	# total genomes	Lineage
Singapore	88.84	406	457	B.6.6
India	8.39	48	572	B.6.6
Malaysia	6.56	4	61	B.6.6
Australia	0.22	2	916	B.6.6

May 2020

Country	Percentage	# B.6.6 genomes	# total genomes	Lineage
Singapore	97.08	166	171	B.6.6
Pakistan	9.09	1	11	B.6.6
India	2.81	40	1426	B.6.6

Supplementary figure 5. Statistics of B.6.6 lineage across different countries between March to May 2020.
